# The effects of musicality on brain network topology in the context of Alzheimer’s disease and memory decline

**DOI:** 10.1162/imag_a_00248

**Published:** 2024-08-05

**Authors:** Anna Maria Matziorinis, Alexander Leemans, Stavros Skouras, Birthe Kristin Flo, Tobias Bashevkin, Stefan Koelsch

**Affiliations:** Institute of Biological and Medical Psychology, University of Bergen, Bergen, Norway; Image Sciences Institute, University Medical Center Utrecht, Utrecht, Netherlands

**Keywords:** neurodegeneration, diffusion MRI, Alzheimer’s disease continuum, music perception, stages of objective memory impairment, structural network topology

## Abstract

Music’s role in modulating brain structure, particularly in neurodegenerative contexts such as Alzheimer’s Disease (AD), has been increasingly recognized. While previous studies have hinted at the potential neuroplastic benefits of musical engagement and training, the mechanisms through which music impacts structural connectivity in neurodegenerative pathways remain underexplored. We aimed to examine the impact of music perception skills, active musical engagement, and musical training on structural connectivity in areas relating to memory, emotion, and learning in individuals with worsening memory impairment, investigating the potential neuroplastic effects of music. Employing diffusion tensor imaging (DTI) based structural connectivity and graph theoretical analysis, we investigated brain topological features in 78 participants aged 42 to 85 with a range of memory impairments. Participants were assessed for musical training, engagement, and perception skills. The study analyzed regional and local network topological metrics to examine the influence of musical activities on graph metrics, while controlling for stages of objective memory impairment (SOMI) and diagnosis, separately. This study aimed to elucidate the effects of musical perception skills, active musical engagement, and musical training on structural connectivity within memory, emotion, and learning-related brain areas in individuals with varying degrees of memory impairment. We found enhanced structural connectivity of the right hippocampus and the right posterior cingulate cortex was associated with stronger local network metrics, such as clustering coefficient and betweenness centrality, with increased music perception skills like melody and beat perception. Musical training specifically impacted the clustering coefficient of the right hippocampus and the node degree of the right mid cingulate gyrus. Active musical engagement influenced the eigenvector centrality of the right hippocampus. Furthermore, musical training was associated with enhanced global metrics, such as global efficiency and characteristic path length. Our study integrates diffusion magnetic resonance imaging (MRI) and graph theoretical analysis to reveal significant effects of musical activities on structural connectivity in key brain regions. The results highlight the potential of musical activities to serve as a non-invasive modulatory tool for cognitive resilience, especially in memory impairment and neurodegeneration contexts. These insights contribute to the understanding of delaying AD onset and aiding early-stage patients through music-based interventions, emphasizing the importance of musical engagement in maintaining cognitive and brain health.

## 1 Introduction

### 1.1 Dementia

Dementia presents a growing public health challenge posing significant societal costs. Alzheimer’s disease (AD), the most prevalent form of dementia, accounts for 60–80% of all cases worldwide (Alzheimer’s Association, 2023). The hallmark features of AD include the accumulation of amyloid-beta plaques and neurofibrillary tangles, which significantly impact cognitive functions and overall brain health. As pathological changes increase, the progression of neuronal and synaptic loss manifests in symptoms like severe memory impairment, language difficulties, disorientation, mood fluctuations, and behavioral and psychological symptoms of dementia. Pharmacological treatments have been predominantly used to alleviate dementia symptoms, with minimal impact to halt disease progression nor to cure AD (Cummings et al., 2021).

AD dementia has been conceptualized as belonging to a continuum of degeneration known as the Alzheimer’s disease continuum, with cognitive decline beginning decades before clinical symptoms become evident (Aisen et al., 2017;Warren et al., 2024). The AD continuum spans from preclinical stages to severe dementia. Recognizing the limitations of current pharmacological treatments, the attention has shifted towards adjunctive treatments that may offer symptomatic relief while potentially slowing disease progression. The earliest symptomatic stage on the continuum is known as Subjective Cognitive Decline (SCD), characterized by subjective reports of cognitive decline relating to memory and executive functioning. SCD has been associated with early AD pathology, with neuroimaging studies indicating elevated levels of AD specific biomarkers, such as amyloid-beta pathology and indices of neurodegeneration (ALFA Study et al., 2018;Jessen et al., 2023). Further on the AD continuum is the objective memory impairment stage, known as Mild Cognitive Impairment (MCI), a prodromal stage of AD marked by noticeable changes, elevated AD-specific biomarkers, indices of neurodegeneration, yet may not include difficulties in performing activities of daily living (Petersen et al., 2013;Vos et al., 2015). Recognizing and addressing these stages through early detection and intervention is essential for improving outcomes for individuals at risk of AD.

One important means to counteract cognitive decline is to bolster cognitive and brain reserve through activities that are cognitively, socially, and physically stimulating (Pettigrew & Soldan, 2019). Musical engagement is such an activity (Cuddy et al., 2015;Särkämö, 2018), and consequently, music-based therapies have shown beneficial effects on global cognition (Fusar-Poli et al., 2018), improvements in episodic memory in late life (Romeiser et al., 2021), and increased quality of life (Ito et al., 2022). Music has the ability to activate most of the brain’s regions and neural networks, strengthening brain connections (Mahmood et al., 2022), especially when listening to preferred music (Carrière et al., 2020), between different regions (Reybrouck et al., 2018). Music can also enhance creativity, increase problem-solving abilities, alleviate stress, and release pleasurable hormones like dopamine and serotonin (Mavridis, 2015;Menon & Levitin, 2005), among other beneficial anti-inflammatory effects (Saifman et al., 2023). Furthermore, for patients with AD dementia, music therapy has emerged as a well-tolerated and safe method, demonstrating efficacy in ameliorating behavioral and psychological symptoms of dementia, cognition, and memory scores, and enhancing social connectedness (Bleibel et al., 2023;Leggieri et al., 2019;Matziorinis & Koelsch, 2022;Moreno-Morales et al., 2020). However, little is known about the neural correlates behind these effects.

### 1.2 Effects of music on structural brain plasticity

The potential of music therapy in alleviating dementia symptoms is increasingly attributed to its influence on stimulating neural plasticity, defined as the brain’s intrinsic capacity to reorganize and form new neural connections in response to learning or novel experiences (Chatterjee et al., 2021;Rodrigues et al., 2010). Music stimulates multiple sensory modalities and cognitive functions, making it a useful tool for studying brain plasticity (Münte et al., 2002;Särkämö et al., 2016;Schlaug, 2015;Wan & Schlaug, 2010). Notably, the brains of older adults have the capacity for neural plasticity (Chaddock-Heyman et al., 2021;Kolb et al., 2003), and music can be used as a cognitive enhancer (Román-Caballero et al., 2018). Neuroimaging studies have highlighted significant structural and functional changes attributed to intensive music training (Groussard et al., 2010;James et al., 2020;Jäncke, 2009;Olszewska et al., 2021;Wan & Schlaug, 2010), yet the precise mechanisms through which music training influences cognitive functions across the life span and how it may serve as a protective factor against cognitive decline also remain unknown.

### 1.3 Effects of musical training and cognitive functions

Recent studies have begun to explore the connection between musical training and cognitive functions. Skills acquired through musical training, such as increased attention, memory, and auditory discrimination, may strengthen non-musical related abilities (Barrett et al., 2013;Miendlarzewska & Trost, 2014;Strait & Kraus, 2011). Furthermore, active musical engagement and musical training may serve as protective factors for enhanced brain health (Matziorinis et al., 2022). Music training may also influence robust structural connectivity, allowing for a buffer against age-related decline and neurological disorders (James et al., 2020). In particular, a study using 6 months of piano training in healthy elderly individuals found that musical training was associated with experience-driven plasticity, stabilizing white matter microstructure in the fornix, a white matter tract involved in memory processing (Jünemann et al., 2022). Another study found that music interventions in healthy older adults not only enhanced cerebellar grey matter but also improved auditory working memory. These results were observed despite normative age-related cerebral atrophy (Marie et al., 2023). These findings underscore music training’s ability to mitigate age-related cognitive decline and further highlighting the potential of music as a tool for cognitive training. Building on previous findings (Cheng et al., 2023;Worschech et al., 2023), our research seeks to address how music affects brain structure and structural connectivity in an aging population with memory decline.

### 1.4 Musical perception and musical memory

In addition to the cognitive benefits conferred by musical training, music perception skills play a critical role in the context of neurodegenerative diseases. Music perception skills, like the ability to recognize familiar tunes, process rhythm and emotions in music, have been studied in relation to neurodegenerative diseases such as AD and Parkinson’s disease. Research indicates that individuals with neurodegenerative diseases often retain musical abilities even when other cognitive functions are impaired (Fang et al., 2017;Petrovsky et al., 2021). Additionally, familiar music interventions have been linked to improvements in self-consciousness and autobiographical memory in individuals with mild to moderate AD. Music recognition and processing in neurodegenerative diseases involve complex neural circuits that may be less affected compared to other cognitive functions, making music a valuable tool for therapy and support in managing these conditions (Golden et al., 2016;Johnson & Chow, 2015).

The preservation of musical memory in individuals with AD, even at advanced stages, is a fascinating phenomenon that highlights the resilience of certain cognitive abilities despite general cognitive decline. Musical memory appears to be partially independent of other memory systems, and in the context of AD, may be partially preserved (Groussard et al., 2019;Moreira et al., 2018). This resilience is believed to be due to the way musical memory is encoded, stored, and retrieved in the brain, which differs from other types of memories. There is evidence that music memory and perception is part of distinct neural networks that are less prone to degradation, such as the pre-ventral supplementary motor area and the anterior cingulate cortex (Cuddy et al., 2015;Jacobsen, Stelzer, Fritz, Chételat, et al., 2015).

Moreover, the relationship between musical abilities and cognitive functions in older adults with MCI offers a promising path for exploration. Previous studies (Jacobsen, Stelzer, Fritz, Chételat, et al., 2015;Petrovsky et al., 2021) have identified modest associations and partially preserved musical abilities in MCI and AD, respectively. These findings open up potential therapeutic strategies, yet the role of music training in preserving or enhancing cognitive functions in MCI and AD remains unclear.

### 1.5 Stages of objective memory impairment

As AD is now recognized to develop along a continuum of degeneration, early detection and investigation of modifiable risk factors becomes imperative to develop effective preventive and therapeutic strategies. Non-invasive assessment tools, such as SOMI, provide a viable pathway to stage memory impairment as it progresses. SOMI staging uses the Free and Cued Selective Reminding Test (FCSRT;Buschke, 1984;Grober & Buschke, 1987), an episodic memory test recommended by the International Working Group as a reliable tool for the assessment of specific cognitive deficits associated with AD (Grande et al., 2018;Teichmann et al., 2017). The FCSRT is structured to optimize the distinction between authentic hippocampal impairments characteristic of AD and memory dysfunctions related to aging, which are often attributed to diminished attention span, suboptimal information processing, and inadequate memory retrieval mechanisms (Grande et al., 2018). As a sensitive measure of change, the FCSRT can be used to track progression of AD. The FCSRT can increase diagnostic accuracy for the development of AD in individuals with MCI (Grande et al., 2018;Grober et al., 2018), and the test can distinguish between frontotemporal dementia and AD (Lemos et al., 2014), as well as suspected AD (Grober et al., 2010;Matias-Guiu et al., 2017). Further, the FCSRT can delineate between retrieval and storage impairments, such as impairments in free recall and total recall, useful for validating disease progression from MCI to AD (Grober et al., 2023). Thus, SOMI categorizes participants into distinct stages (0–4), simultaneously accommodating for progressive alterations in memory retrieval and storage impairments characteristic of the Alzheimer continuum. Refer toTable 1for SOMI stage classification.

**Table 1.** Characterizing SOMI stages using FR and TR scores with associated class of memory impairment.

SOMI stage	Free recall scores	Total recall scores	Class of memory impairment
0 No Memory Impairment	>30	>46	None detected by pFCSRT + IR task
1 Subtle Retrieval Impairment	25–30	>46	Free Recall declines at a constant rate. Storage is preserved.
2 Moderate Retrieval Impairment	20–24	>46	The rate of free recall decline doubles. Executive dysfunction accelerates. Storage is preserved.
3 Subtle Storage Impairment	any	45–46	Cueing fails to normalize total recall.
4 Significant Storage Impairment Compatible with Dementia	any	33–44	Intellectual decline accelerates, heralding activities of daily living impairment.
5 Atypical	<20	>46	Retrieval impaired but storage unimpaired.

pFCSRT + IR = picture version of the Free and Cued Selective Reminding Test with Immediate Recall; SOMI = Stages of Objective Memory Impairment. A subset of participants (atypical) does not meet the SOMI criteria as summarized in the table (FR < 20 and TR > 46). Their retrieval is impaired, but storage is unimpaired.

### 1.6 The present study

The present study investigates the influence of musical behaviors and music perception skills on structural brain plasticity and its potential modulatory effects on cognitive functions in the context of AD dementia and progressive memory decline. Through the analysis of diffusion tensor imaging (DTI) based structural connectivity and network topological metrics across key brain regions involved in memory, learning, and emotional processing, this study aims to explore the mechanisms underlying the effects of music-based interventions and their potential to bolster cognitive health and resilience in individuals with progressive memory decline.

Using DTI-based structural connectivity alongside applied graph theoretical analysis, we evaluated regional and local structural network metrics with brain areas related to the Papez circuit. The Papez circuit is a medial limbic circuit known for its role in learning, memory, and emotion, making it a focal point for assessing the impact of musical engagement on brain structure (Aggleton et al., 2016). To measure music’s influence on structural network attributes in participants with memory decline, our study incorporates the SOMI system (SOMI;Grober et al., 2018,^2021^).

By examining the nuanced interplay between musicality and cognitive decline, we aim to shed light on potential neuroprotective effects of music and its implications for cognitive resilience. Our investigation is structured around three core hypotheses, each designed to explore different facets of how music perception and cognitive abilities interact in the progression of AD.

Firstly, we posit that, as SOMI stages progress, there will be a corresponding decline in music perception skills, similar to the decrease observed in neurocognitive memory test performance. We aim to investigate whether the deterioration of music perception skills parallels the escalation of cognitive impairment.

Secondly, we hypothesize that participants with increased musicality, quantified by elevated scores on the Goldsmiths Music Sophistication Index (Gold-MSI;Müllensiefen et al., 2014) and intrinsic music perceptual skills, measured by the mini-version of the Profile of Music Perception skills (mini-PROMS;Law & Zentner, 2012;Zentner & Strauss, 2017), will exhibit greater hippocampal-based brain reserve (HBBR) (Yildirim et al., 2023), a macrostrutural indicator of structural integrity and a well-researched proxy for brain atrophy in AD research (Vemuri & Jack, 2010). Further, we investigate whether DTI-based indices of microstructural change, such as fractional anisotropy (FA) and mean diffusivity (MD), will correlate to music perception skills and musical behaviors.

Thirdly, we hypothesize that increased musical engagement, musical training, and music perception skills will be associated with enhanced regional and local structural network topological metrics.

## 2 Material and Methods

### 2.1 Participants

Seventy-eight Norwegian-speaking participants with a mean age of 71.06 years of age (*SD*= 9.85) and a mean of 14.14 years of education (*SD*= 3.21) were included in the study and stratified into SOMI stages 0 through 4; seeTable 2for demographic statistics andTable 3for diagnostic classification.

**Table 2.** Descriptive statistics and demographics across SOMI stages (*N*= 78).

	SOMI-0	SOMI-1	SOMI-2/3	SOMI-4
N	n = 25	n = 18	n = 18	n = 17
Age	65.32 (10.72)	69.56 (10.42)	75.67 (5.54)	76.24 (6.34)
gender (m/f)	9/16	9/9	8/10	9/8
years of education	14.92 (3.29)	13.67 (2.72)	13.00 (3.38)	14.71 (3.24)
handedness (right)	25	18	17	17
medication type (% yes)				
Alzheimer’s	0.0	0.0	0.0	29.4
sleep	4.0	5.6	5.6	0.0
allergy	16.0	22.2	5.6	0.0
blood pressure	28.0	50.0	44.4	41.2
metabolism	12.0	5.6	11.1	11.8
anxiety	0.0	0.0	5.6	0.0
antidepressants	0.0	0.0	11.1	0.0
cholesterol	12.0	27.8	16.7	29.4
other	36.0	61.1	33.3	29.4
smoking (% yes)	4.00	5.6	0.0	5.9
smoking (previously)	24.0	22.2	11.1	17.6

Listed values included sample size (n), mean age and standard deviation, gender (sex assigned at birth), either Male or Female, mean years of education, and standard deviation. Handedness is listed as those who are right-handed. Medication type listed in terms of affirmative use of medication type (percentage of those who replied yes to using medication).

**Table 3.** Demographic and clinical characteristics of study participants by diagnostic category.

	Sub-SCD	SCD	P-MCI	MCI	AD
N	n = 22	n = 26	n = 14	n = 6	n = 10
Age	69.45 (9.03)	68.08 (11.63)	74.57 (8.64)	76.00 (6.99)	74.50 (6.98)
gender (m/f)	7/15	13/13	7/7	2/4	6/4
years of education	14.82 (3.70)	14.31 (2.57)	11.86 (2.60)	14.17 (2.86)	15.40 (3.57)
handedness (right)	22	25	13	6	10
medication type (% yes)					
Alzheimer’s	0.0	0.0	0.0	0.0	50.0
sleep	9.1	3.8	0.0	0.0	0.0
allergy	4.5	19.2	21.4	0.0	0.0
blood pressure	40.9	34.6	50.0	33.3	40.0
metabolism	22.7	0.0	7.1	0.0	20.0
anxiety	0.0	0.0	7.1	0.0	0.0
antidepressants	9.1	0.0	0.0	0.0	0.0
cholesterol	22.7	19.2	21.4	16.7	20.0
other	54.5	30.8	50.0	33.3	20.0
smoking (% yes)	9.1	3.8	0.0	0.0	10.0
smoking (previously)	27.3	11.5	28.6	16.7	10.0

Categories include: Sub-SCD: Sub-threshold SCD, individuals who did not meet both the criteria by self-report and informant report of a score > 7 on the MyCog and TheirCog subscales of the SCD-Q questionnaire; SCD: Subjective Cognitive Decline; individuals meeting the criteria for SCD using the SCD-Q; P-MCI: Probable MCI, currently undergoing assessment by a physician; MCI: individuals diagnosed with Mild Cognitive Impairment; AD: individuals diagnosed with Alzheimer’s Disease. Listed values included sample size (n), mean age and standard deviation, gender (sex assigned at birth), either Male or Female, mean years of education, and standard deviation. Handedness is listed as the number of individuals who are right-handed. Medication type listed in terms of affirmative use of medication type (i.e., percentage of those who replied yes to using medication). Smoking type listed as current smokers or previous smokers (i.e., percentage of those who replied yes for current smoking or previous smoking).

The inclusion criteria specified that participants had to be: (1) proficient in Norwegian or have Norwegian as their first language, (2) reside independently at home, (3) report subjective memory decline or complaints, (4) be capable of providing written informed consent, (5) be able to undergo brain scanning, and for those diagnosed with early-phase AD or MCI, (6) the presence of an accompanying caregiver was required, who would also provide informed consent.

Exclusion criteria included other diagnoses of (1) severe psychiatric disorders, (2) neurological diseases, (3) sensory disorders, notably deafness or auditory impairments, (4) vascular disorders or complications, that is, history of heart disease, heart attack, heart surgery, or stroke, (5) diagnoses of other dementia subtypes, for example, Lewy body dementia, fronto-temporal dementia, or vascular dementia, (6) a history of traumatic brain injury, (7) claustrophobia, (8) implants incompatible with MR scanning, or (9) participants meeting criteria for SOMI stage 5 (seeTable 1).

All participants signed written informed consent. Ethics approval was awarded by the Regional Committees for Medical and Health Research Ethics (REC-WEST: reference number 2018/206).

#### 2.1.1 Stages of objective memory impairment (SOMI)

Participants were stratified into five stages of SOMI, ranging from SOMI-0 to SOMI-4. Categorization was based on performance of the picture version of the Free and Cued Selective Reminding task, which includes immediate Recall (pFCSRT + IR). The procedure for the FCSRT starts with an encoding phase. During this phase, participants were shown four groups of images, each containing four items (e.g., a picture of an owl). Each picture is paired with a category cue (e.g., the cue is “animal”). Following this, participants underwent a test to gauge their immediate recall of these image sets under a controlled learning environment. The entire task was divided into three trial runs, each interrupted by a task designed to divert attention. Participants subtracted three from 100 continuously for 30 seconds. The test outcomes were measured in terms of free recall (FR; maximum score of 48) where items are recalled without the aid of a category cue, cued recall (CR) which measures items recalled with the assistance of a cue, and total recall (TR; maximum score of 48) which measures the combination of both FR and CR scores. After a certain period, typically after 20–30 minutes, participants are retested to evaluate their delayed free recall and delayed cued recall scores revealing the participants’ ability to retrieve information after a delay showing their long-term memory retention and retrieval capabilities.

#### 2.1.2 Subjective cognitive decline questionnaire (SCD-Q)

The Subjective Cognitive Decline Questionnaire (SCD-Q; (Rami et al., 2014) assesses self-reported cognitive decline. The questionnaire is split into two segments: the MyCog section, completed by the individual, and the TheirCog section, completed by an informant, usually a close relative. The SCD-Q includes 24 questions addressing perceived declines in memory, language, and executive functions over the past 2 years. Scores range from 0 to 24, with higher scores indicating greater cognitive decline. A score of seven or higher on both the MyCog and TheirCog sections indicates SCD. Sub-threshold SCD is indicated when one section, either MyCog or TheirCog, scores above seven, but this score is not confirmed by the other section.

#### 2.1.3 Mini-mental state examination Norwegian revised (MMSE)

The Mini-Mental State Examination (MMSE-Norwegian Revised version (Folstein et al., 1975;Strobel & Engedal, 2008) is used to screen for cognitive impairment and asses mental status. As the most widely used screening tool for dementia (Michelet et al., 2018), the MMSE is a brief global assessment of cognitive status with 30 items including orientation, attention, memory, language, and visuospatial skills, culminating in a total MMSE score. Scores range from 0 to 30, with scores under 17 being typical of moderate-to-severe dementia (Schultz-Larsen et al., 2007). A maximum score of 30 is indicative of healthy cognitive status.

#### 2.1.4 Consortium to establish a registry for Alzheimer’s disease (CERAD) word list memory test

The Word List Memory test from The Consortium to Establish a Registry for Alzheimer’s Disease (CERAD;Morris et al., 1989) was used to evaluate verbal memory. In this test, participants are shown 10 unrelated words, which they need to remember and recall across three separate trials. The total score for these trials ranges from 0 to 30. Additionally, the test includes a delayed free recall component, where participants recall the words after a period of time, scored from 0 to 10, and a recognition part of the test, where participants are presented with 20 words, which is a mix of the original 10 words with 10 new words. The task is to identify the original words, with scoring from 0 to 20. This comprehensive approach helps in assessing different aspects of verbal learning and memory.

#### 2.1.5 Goldsmiths musical sophistical index (Gold-MSI)

We measured musical behaviors using the Goldsmiths Musical Sophistication Index (Gold-MSI; (Müllensiefen et al., 2014), v1.0, 11 October 2012 (https://media.gold-msi.org/test_materials/GMS/docs/gms_documentation_en.pdf). The Gold-MSI is a self-assessment tool developed to quantify self-reported musical proficiencies, achievements, musicality, and related musical behaviors of the general populace. This index captures individual variations in active participation, music training, and emotional responsiveness to music, among other factors. The Gold-MSI is a well-validated questionnaire that measures musical experience across five quantifiable subscales: active engagement, perceptual abilities, emotions, singing abilities, and musical training. We selected items using the Gold-MSI questionnaire configurator (https://shiny.gold-msi.org/gmsiconfigurator/). To view the complete questionnaires refer to theSupplementary Materials.

Each item was scored on a seven-point Likert scale from “absolutely disagree” to “absolutely agree,” with higher scores reflecting higher levels of musical behavior. Four of the 19 items were negatively coded to ensure construct validity and reliability. Mean subscale scores were calculated using the sum of the subscale items and then further divided by the total number of questions for that particular subscale.

In the analysis of the results of the Gold-MSI, there were four negatively coded items. These items were reverse-scored before conducting any statistical analyses to ensure that higher scores consistently reflected higher levels of musical sophistication across all items. This approach aligns with best practices for handling Likert-scale data, ensuring that all items contribute positively and uniformly to the derived subscale scores.

We used two full subscales (active engagement and musical training) and selected questions from the emotions subscale. The active engagement (AE) subscale is defined as a person’s musical engagement level, including reading, writing, internet activities relating to music, music listening, and total income spent on musical materials and shows. The musical training (MT) subscale is defined as the level of musical dedication and practice (including both formal and informal training) associated with time spent on musical training (e.g., “how many peak hours per day on musical practice spent or the amount of musical training in years”). The MT subscale also includes the number of instruments played, including voice, as well as any background of music theory studied. The questions used for the emotions subscale evaluated an individual’s ability to articulate emotions conveyed by music, use of music for motivation, and the propensity to get chills when listening to music.

#### 2.1.6 Mini-version of the profile of music perception skills (Mini-PROMS)

The original version of the Profile of Music Perception Skills (PROMS) (Law & Zentner, 2012) is a comprehensive diagnostic tool designed to evaluate musical aptitude in the general population. The PROMS allows for quantifying music perception skills across four main categories, including tonal, temporal, dynamic, and textural features. The test stands out as an efficient, versatile, and reliable tool for assessing musical aptitude.

For brevity, we used the mini-version of the PROMS, a shortened version of the test battery that has been validated for online administration (Zentner & Strauss, 2017). The test duration is approximately 20–25 minutes in length, where participants are evaluated across four critical musical dimensions including melody, tuning, rhythm, and tempo perception, culminating in a total mini-PROMS score. The test involves discerning between two repetitive stimuli and a third distinct stimulus. This approach not only tests basic recognition skills, but also challenges the participant’s ability to perceive and discern musical subtleties within distinct musical elements.

The structure of the mini-PROMS includes “same-different” judgment tasks, where the items of the questionnaire were rated on a five-point Likert scale. The scale ranges from “definitely same,” “maybe same,” “do not know,” “maybe similar,” to “very similar.” There was also the inclusion of a “do not know” option which provides insight into the participant’s confidence level. Each stimulus was repeated twice using a reference and repetition stimuli, and subsequently played a third time as the comparison stimuli. Before each trial block, participants listened to a practice trial to ensure they understood the task. Each subtest consisted of 8 to 10 trials per musical dimension with an equal number of same-correct and different-correct answers per subtest. The neutral response (i.e., “do not know”) was scored as 0 points, and the correct “maybe” responses were scored as 0.5 points, with the maximum score for correct responses being scored as 1 point. All stimuli were presented at the same volume in a private and quiet room.

#### 2.1.7 Finger-tapping task (FTT)

We employed a finger-tapping task (FTT;Reitan & Davison, 1974), a simple motor task that evaluates the tapping speed of the fingers (using the right and left index fingers) and the time between each tap. The FTT has been used as a tool to assess cognitive function and motor skills in individuals with AD and disorders related to cognitive decline, as finger motor skills have been found to be impaired in patients with AD and MCI, suggesting fine motor functioning declines even before the onset of dementia (Roalf et al., 2018;Suzumura et al., 2016,^2021^).

Participants were instructed to tap their index finger with their dominant and non-dominant hand as rapidly and accurately as possible using the space bar on a keyboard across five blocks interspersed by short breaks between blocks. The finger-tapping task was programmed in-house using PsychoPy, and left and right-hand tapping measures were subsequently extracted, including the generation of a computed combined mean.

### 2.2 Brain data

#### 2.2.1 MRI acquisitions

Participants underwent magnetic resonance imaging (MRI) using a 3-T GE Discovery MR750 brain scanner (General Electric Medical Systems, Milwaukee, WI, USA) equipped with a 32-channel head coil. Anatomical images were obtained using a sagittal 3D T1-weighted fast spoiled gradient-echo (FSPGR) sequence with parameters: repetition time = 6.9 ms, echo time (TE) = 3.0 ms, slice thickness = 1.0 mm, in-plane field of view (FOV) = 25.6 by 25.6 cm^2^, matrix size = 256 by 256, flip angle = 12°, and inversion time = 450 ms. Acquisition duration was 9 minutes.

Diffusion-weighted images were captured using a single-shot spin-echo echo-planar imaging sequence. The parameters included: 60 axial slices with a thickness of 2.4 mm (no inter-slice gap), FOV = 220 by 220 by 220 mm^3^, acquisition matrix = 128 by 128, reconstructed voxel dimensions = 1.72 by 1.72 by 2.4 mm^3^, repetition time = 14,000 ms, TE = 93 ms, flip angle = 90°, b-value = 1,000 s/mm^2^applied in 30 non-collinear diffusion directions, and six non-diffusion-weighted scans (b = 0 s/mm^2^). Acquisition duration was 8 minutes.

For entertainment purposes, participants were shown visual stimuli comprising natural landscapes during the structural imaging sessions, accompanied by instrumental music. Participants picked their type of music out of a selection of six genres: classical, jazz, world, pop, rock, and folk, as curated by the research team.

#### 2.2.2 MRI data analysis

Structural T1-weighted MR images were defaced for anonymity, visually quality inspected, and individual structural T1-weighted MR images further underwent cortico and subcortical segmentation and parcellation procedures (FreeSurfer v7.3.2,https://surfer.nmr.mgh.harvard.edu/). The pipeline includes normalization, non-brain tissue removal, Talairach transformations, segmentation, and delineation of grey and white matter boundaries as described byFischl (2004). The cortical regions were parcellated with the Desikan-Killiany atlas (Desikan et al., 2006) which included 34 cortical and 9 subcortical brain regions bilaterally.

#### 2.2.3 Hippocampal-based brain reserve

FreeSurfer subcortical segmentation statistics was used to extract hippocampal volumes and intracranial volume. HBBR was calculated through the summation of the bilateral hippocampal volumes (left and right) and further divided by the total estimated intracranial volume (Van Loenhoud et al., 2018). These values were then standardized and orthogonalized to age (in years) using the spm_orth function in SPM12 (Statistical Parametric Mapping) (http://www.fil.ion.ucl.ac.uk/spm).

$$HBBR=\frac{V_{\text{Right hippocampus}}+V_{\text{Left hippocampus}}}{V_{\text{Total intra cranial volume}}}$$

#### 2.2.4 Diffusion tensor imaging and fiber tractography

DTI analysis was conducted using ExploreDTI (v4.8.6;Leemans et al., 2009) within MATLAB vR2022a (Mathworks Inc., Natick, MA, United States), with DTI-focused automated procedures implemented through the ExploreDTI graphical user interface. DTI preprocessing of diffusion weighted images included: (a) correcting for signal intensity drift and Gibbs ringing artifacts, (b) correcting for subject motion and eddy current induced geometrical distortions (Leemans & Jones, 2009), in addition to susceptibility distortions on co-registered T1-weighted MR images, (c) adjusting B-matrices with appropriate reorientations, and modulating required signal intensities using a Jacobian determinant of the spatial transformation (Jones & Cercignani, 2010;Leemans & Jones, 2009). Diffusion tensor estimation used non-linear regression (Basser & Jones, 2002;Basser et al., 1994;Veraart et al., 2013), and subsequent whole-brain deterministic tractography was performed based on the following parameters: Fractional anisotropy (FA) tracking threshold of 0.2, angle threshold of 30°, step size of 1 mm, seed points sampled at a uniform resolution of 2 by 2 by 2 mm^3^, and using a fiber tract length range of 50–500 mm. SeeFigure 1for graphical overview of the processing pipeline.

**Fig. 1.**
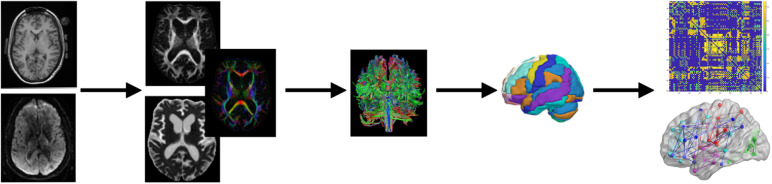
Graphical overview of the processing pipeline for DTI-derived structural connectivity measures. In sequence from left to right, the steps include structural T1-weighted preprocessing using FreeSurfer, DTI image corrections including motion, eddy currents, and EPI correction, deterministic whole brain tractography, application of the AAL atlas template to identify each pair of nodes, and network analysis resulting in graph construction using FA streamlines into binary matrices.

#### 2.2.5 Diffusion metrics

The most commonly used measures in diffusion weighted imaging are fractional anisotropy and mean diffusivity (MD), which provide insights into the integrity of white matter. In our investigation, we focused on FA and MD for our analyses. Refer toSupplementary Materialsfor derivation of DTI metrics.

#### 2.2.6 Network analysis of structural connectivity measures

For network analysis and visualization of complex brain networks, we used the Brain Connectivity Toolbox (BCT) byRubinov and Sporns (2010)to obtain graph theoretical metrics. To construct individual DTI-based connectivity matrices, the Automated Anatomical Labeling (AAL) atlas (Tzourio-Mazoyer et al., 2002) was used to partition the brain into 90 regions. The AAL atlas is a widely used atlas for deriving nodes in graph theoretical analysis. FA values were extracted from all fibers connecting each pair of regions, serving as edges in the network analysis. The cerebellum was excluded, resulting in a total of 90 nodes (*N*= 90) comprising 78 cortical and 12 subcortical nodes.

PASS filters were used to weight the matrices by the number of FA streamlines connecting node*i*to node*j.*PASS filters connect pathways in a sequential manner. For example, pathway A is connected to pathway B is connected to pathway C, rather than pathway A connecting to pathway C, disregarding pathway B. These weighted matrices were then combined into a 90 by 90 adjacency (connectivity) matrix. All self-connections were excluded from the analysis by setting the diagonal values to 0. For metrics requiring connection lengths, these lengths were calculated using the weight_conversion.m (“lengths”) function in the BCT toolbox.

Graph theoretical metrics were calculated based on all 90 nodes defined using the AAL atlas. Measures of global or regional connectivity such as global efficiency, characteristic path length, and nodal density were determined across all 90 nodes. Further, local metrics were calculated including clustering coefficient, eigenvector centrality, betweenness centrality, and node degree.

#### 2.2.7 Definition of global and local metrics

A local metric evaluates the property of a single node, while global metrics gauge the overall property of the network, often defined as the mean of local metrics (Cozzo et al., 2018).

We employed local network metrics recognized as pivotal indicators of network structure, namely the clustering coefficient, eigenvector centrality, betweenness centrality, and node degree. The clustering coefficient measures the tendency of network elements to cluster together, shedding light on the network’s segregation. Eigenvector centrality gauges the influence of a node within the network, highlighting critical areas of information flow. Node degree quantifies the number of direct links a node possesses, unveiling data regarding network density and potential communication pathways. Betweenness centrality assesses the degree to which a node is situated on paths between other nodes, providing insight into the potential for control or influence within a network. These metrics were examined in conjunction with cortical white matter regions linked to the Papez circuit (Forno et al., 2021), a medial limbic circuit connected to learning, memory, and emotional processing. The circuit comprised several key structures, namely the hippocampus, fornix, mammillary bodies, anterior thalamic nuclei, and cingulate gyrus. Together, these structures work together to regulate emotional responses, form and consolidate memories, and integrate sensory information (Aggleton et al., 2016).

Regarding global metrics of interest, we used characteristic path length, global efficiency, and node density. The characteristic path length is defined as the average shortest path length in the network, representing the average number of steps along the shortest paths for all possible pairs of network nodes. Global efficiency embodies the efficacy of information exchange on a broad scale in a network, measuring how efficiently the network as a whole exchanges, integrates, and distributes information regionally. Node density signifies the average of the node degrees across all nodes in a network, representing an overall level of interconnectivity. A higher node density implies that nodes have more neighbors, facilitating the transfer of information between nodes.

#### 2.2.8 Regions of interest analysis

Regions-of-interest analysis was conducted encompassing several key structures of the Papez circuit which included the bilateral thalami, hippocampi, anterior, middle, and posterior cingulate, and parahippocampal region which itself includes the medial portion of the subiculum, entorhinal cortex, transentorhinal cortex, and parahippocampal neocortex. SeeFigure 2for visualization of Papez region that could be constructed using the AAL atlas (version 1).

**Fig. 2.**
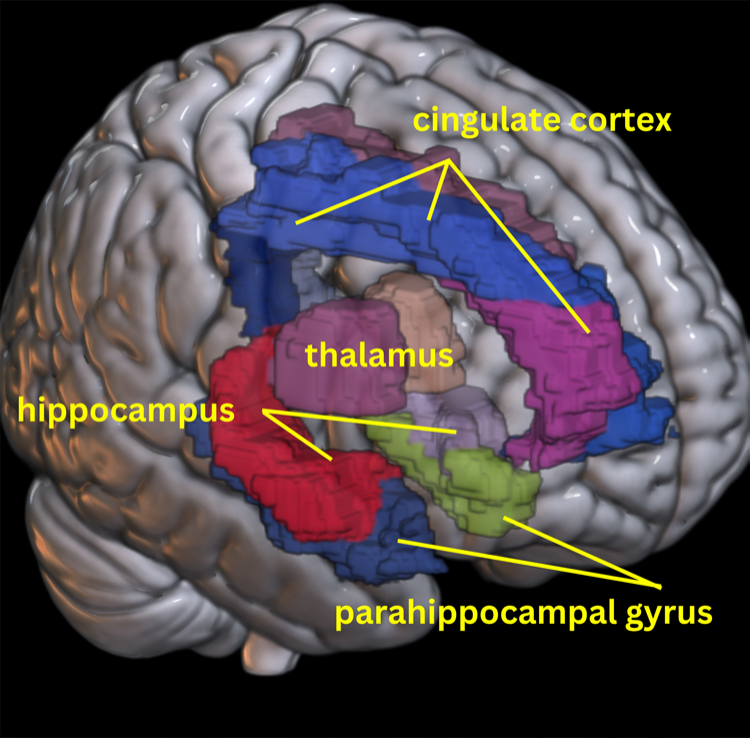
Regions of interest. Displays the 12 regions of interest selected using the AAL atlas (version 1), including bilateral regions: thalami (muted raspberry and tan), hippocampi (red and lilac), anterior (magenta and blue), middle (dusty rose and light blue), and posterior cingulate (dark blue and grey) cortex, and parahippocampal gyrus (navy blue and light green) which includes the medial portion of the subiculum, entorhinal cortex, transentorhinal cortex, and parahippocampal neocortex.

### 2.3 Statistical analysis

Statistical analyses were performed using SPSS software (IBM Corp. Released 2020. IBM SPSS Statistics for Windows, Version 28.0. Armonk, NY: IBM Corp) and JASP (JASP Team (2023). JASP (Version 0.17.3)). Our investigation categorized musical behaviors and skills into three broad categories: music perception skills (assessed by the mini-PROMS), musical engagement, and musical training (both evaluated through the Gold-MSI along with selected questions about emotional experience).

Prior to comparative analyses, musical measures (Gold-MSI and mini-PROMS) were standardized into z-scores to facilitate direct comparisons with brain ROIs. Higher z-scores thus reflected superior performance on music tests, enabling a uniform evaluation metric across different measures.

Next, associations were made between the scores from the Gold-MSI, mini-PROMS, measures of brain reserve (indicated by HBBR), and a proxy for cognitive reserve (years of education). Additionally, DTI metrics of microstructural change, such as FA and MD, were correlated with Gold-MSI, mini-PROMS, and HBBR. All association analyses were evaluated using Spearman’s rank correlations. This choice was motivated by the non-parametric nature of our data, providing insights into the strength and direction of these relationships without assuming linear correlations.

To examine the impact of music perception skills across different stages of memory impairment, we employed a one-way Analysis of Covariance (ANCOVA), with musical measures as dependent variables. This analysis incorporated SOMI stages and gender as fixed factors, while adjusting for age and years of education as covariates. Post hoc analyses, including estimated marginal means and pairwise comparisons with Bonferroni correction, were conducted to refine our understanding of the relationships, ensuring that the influence of confounding factors was minimized.

Similarly, to examine the impact of musical behaviors on different stages of memory impairment, we employed an ANCOVA, with musical measures as dependent variables. Each comparison included a DTI-derived graph theoretical metric for specific ROIs as the dependent variable. The models accounted for SOMI stage and gender as fixed factors, with age and years of education as covariates. To ensure more equitable group sizes, SOMI stages 2 and 3 were combined, resulting in a total of four distinct stages of memory decline for analysis. Post-hoc Bonferroni corrections were applied to account for multiple comparisons.

## 3 Results

### 3.1 Impact of music perception skills on SOMI

First, we investigated the influence of music perception skills, as measured by beat, tempo, melody, tuning, and the total mini-PROMS score, across different SOMI stages. Refer toTable 4for means and standard deviations of the music-related questionnaires. Our analysis included participants categorized into various SOMI stages to discern any potential variances in music perception abilities attributable to the progression of memory impairment. However, no significant differences in music perception skills across the SOMI stages were found, encompassing beat, tempo, melody, tuning, and total mini-PROMS scores (seeFig. 2for details and full statistical analyses inSupplementary Materials).

**Table 4.** Neurocognitive, brain reserve, music engagement, and perception skills by SOMI stages (*N*= 78).

	SOMI-0	SOMI-1	SOMI-2/3	SOMI-4
Sample size	n = 25	n = 18	n = 18	n = 17
**Cognition**				
MMSE total	28.44 (1.69)	28.11 (2.06)	27.05 (2.16)	22.12 (4.05)
**Memory**				
FCSRT-IR				
total free recall	34.16 (2.66)	27.17 (1.82)	20.50 (3.71)	9.17 (9.36)
total recall	47.92 (0.28)	47.72 (0.46)	47.17 (1.04)	28.25 (16.64)
delayed free recall	12.72 (1.49)	10.94 (1.47)	8.39 (2.30)	3.42 (4.34)
delayed cued recall	3.20 (1.53)	5.00 (1.46)	7.44 (2.20)	6.25 (3.79)
Word List Memory Test				
word learning total	21.00 (3.06)	19.89 (4.36)	16.67 (4.16)	10.76 (4.74)
word learning delayed recall	7.76 (1.36)	6.61 (1.94)	4.72 (2.02)	1.75 (1.65)
word learning recognition	19.84 (0.37)	19.56 (0.78)	18.56 (1.25)	15.94 (2.38)
**Music Engagement**				
Gold-MSI				
active engagement	3.18 (1.24)	3.35 (0.99)	3.96 (1.04)	3.21 (1.30)
musical training	2.19 (1.24)	2.46 (1.19)	2.57 (1.21)	2.37 (1.03)
Emotions	4.70 (1.49)	4.84 (1.20)	4.90 (1.37)	4.47 (1.34)
**Music Perception Skills**				
mini-PROMS				
Melody	5.22 (1.60)	5.25 (1.66)	5.33 (1.69)	4.59 (1.95)
Tuning	4.32 (1.42)	4.48 (1.62)	4.08 (1.26)	3.91 (0.89)
Beat	4.80 (1.54)	4.22 (1.33)	4.47 (1.21)	4.71 (1.48)
Tempo	5.12 (1.94)	5.39 (1.48)	5.14 (1.66)	4.44 (1.43)
mini-PROMS total score	19.46 (4.28)	19.44 (4.95)	19.03 (3.99)	17.65 (4.53)
**Motor Skills**				
Finger-tapping task				
left-hand	48.36 (7.68)	45.89 (7.32)	44.81 (6.94)	39.00 (11.83)
right-hand	52.81 (7.38)	48.66 (7.05)	45.49 (8.04)	43.41 (16.16)
combined average	50.58 (6.95)	47.28 (6.56)	45.15 (6.58)	41.21 (13.76)
**Macrostructural: Brain Reserve**				
HBBR	0.29 (0.74)	0.43 (0.68)	-0.10 (0.91)	-0.58 (0.77)

Mini-mental state examination (MMSE); Goldsmiths sophistication index (Gold-MSI); Mini-version of the profile of music perception skills (mini-PROMS) including four subscales; melody, tuning, beat, tempo, and Proms total score; finger-tapping task (FTT), including left-hand, right-hand, and combined average score, Hippocampal-based brain reserve (HBBR). The listed values are means and standard deviations.

### 3.2 Protective effects of brain reserve on musical sophistication and music perception abilities

Next, we examined the relationship between music perception skills and hippocampus-based brain reserve (HBBR, orthogonalized for age) using Spearman’s rho correlations. HBBR showed no significant correlations with emotional engagement (*ρ*= 0.045,*p*= 0.716), active engagement (*ρ*= -0.043,*p*= 0.725), musical training (*ρ*= -0.095,*p*= 0.442), or any of the music perception skills measured by the mini-PROMS (Tuning, Beat, Tempo, Melody, and total mini-PROMS score), with*ρ*values ranging from -0.028 to 0.081 and*p*> 0.05 for all. This suggests that the structural brain differences implied by brain reserve are not significantly associated with music perception abilities or levels of musical engagement in this sample. Refer toFigure 3for violin plots displaying memory, HBBR, and mini-PROMS total scores across SOMI stages.

**Fig. 3.**
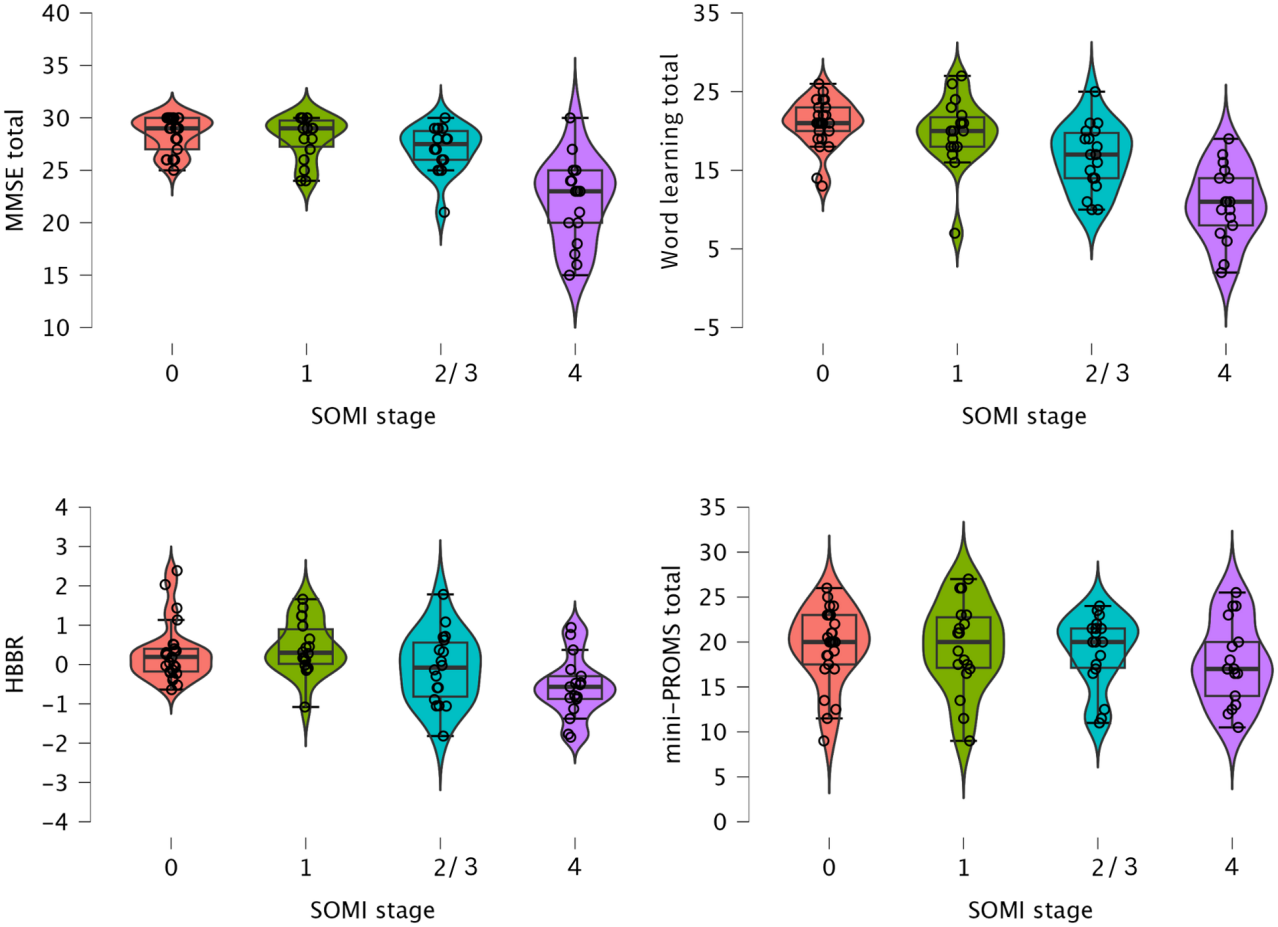
Displays violin plots of memory, brain reserve, and music perception skills across SOMI stages. Displayed are the total scores on the MMSE and Word list memory test along with hippocampal-based brain reserve (HBBR), and the total scores on the mini-PROMS. SOMI stages 0–4 correspond to SOMI stage 0, 1, the combined stages 2 and 3, as well as SOMI stage 4.

### 3.3 Microstructural investigation on musical sophistication and music perception abilities

As microstructural changes typically precede macrostructural changes, we examined the relationship between FA and MD metrics of the left and right hippocampus and musical measures using Spearman’s rho correlations. Both FA and MD showed no significant correlations with music perception abilities nor Gold-MSI subscales. However, MD of the left hippocampus showed a significant correlation to HBBR (*ρ*= -0.453,*p*= < 0.001).

### 3.4 Impact of musical sophistication and music perception abilities on local network topology

Investigating the impact of music perception skills using the mini-PROMS on local graph metrics within the regions of interest, our ANCOVA analyses, controlling for SOMI stage and gender as fixed factors and age and years of education as covariates, revealed significant findings. Refer toTable 5for significant findings. Further ANCOVA analyses using diagnosis and gender as fixed factor, and age and years of education as covariates showed similar significant findings. Refer toSupplementary Materials.

**Table 5.** Effects of music perception scores on local network metrics.

Brain region	Graph metric	Measure	*df*	*F* value	*p* value	Partial * η ^2^*	Effect size	Survives Bonferroni correction
Right Hippocampus	Clustering Coefficient	Melody	1, 67	11.287	**0.001**	0.144	Large	**Yes**
Left Parahippocampal Gyrus	Clustering Coefficient	Melody	1, 67	4.494	0.038	0.063	Small-Moderate	No
Left Parahippocampal Gyrus	Clustering Coefficient	Beat	1, 67	6.295	0.015	0.086	Moderate	No
Left Parahippocampal Gyrus	Clustering Coefficient	Total Score	1, 67	7.946	0.006	0.106	Moderate	No
Right Parahippocampal Gyrus	Clustering Coefficient	Melody	1, 67	5.566	0.021	0.077	Moderate	No
Right Parahippocampal Gyrus	Clustering Coefficient	Beat	1, 67	5.094	0.027	0.071	Moderate	No
Right Posterior Cingulate Gyrus	Clustering Coefficient	Melody	1, 67	10.986	**0.001**	0.141	Large	**Yes**
Right Posterior Cingulate Gyrus	Clustering Coefficient	Beat	1, 67	4.402	0.040	0.062	Small-Moderate	No
Right Posterior Cingulate Gyrus	Clustering Coefficient	Total Score	1, 67	8.038	0.006	0.107	Moderate	No
Right Hippocampus	Eigenvector Centrality	Melody	1, 67	5.992	0.017	0.082	Moderate	No
Right Posterior Cingulate Gyrus	Eigenvector Centrality	Total Score	1, 67	4.744	0.033	0.066	Small-Moderate	No
Right Parahippocampal Gyrus	Eigenvector Centrality	Beat	1, 67	4.898	0.030	0.068	Small-Moderate	No
Right Parahippocampal Gyrus	Betweenness Centrality	Melody	1, 67	4.358	0.041	0.061	Small to Moderate	No
Right Hippocampus	Betweenness Centrality	Tuning	1, 67	7.312	0.009	0.098	Moderate	No
Right Hippocampus	Betweenness Centrality	Beat	1, 67	9.453	**0.003**	0.124	Moderate	**Yes**
Right Hippocampus	Betweenness Centrality	Tempo	1, 67	4.124	0.046	0.058	Small	No
Right Hippocampus	Betweenness Centrality	Total Score	1, 67	6.546	0.013	0.089	Moderate	No
Right Mid Cingulate Gyrus	Node Degree	Melody	1, 67	4.401	0.040	0.062	Small-Moderate	No
Right Mid Cingulate Gyrus	Node Degree	Total Score	1, 67	4.664	0.034	0.065	Small-Moderate	No

Table 5presents ANCOVA findings on the influence of music perception skills on network metrics across selected brain regions. Data include the targeted brain region, specific network metric, measure type, degrees of freedom (*df*),*F*value,*p*value, partial eta squared (*η²*) for effect size, and the status of Bonferroni correction survivability. Bold refers to findings that survived bonferroni correction.

The impact of musical sophistication, such as musical training, active engagement with music, and emotional experiences, on local graph metrics within the regions of interest is examined. Our ANCOVA analyses, controlling for SOMI stage and gender as fixed factors, and age and years of education as covariates, revealed significant findings. Refer toTable 6for significant results. Similarly, we conducted further ANCOVA analyses controlling for diagnosis and gender as fixed factors, and age and years of education as covariates, corroborating findings and revealing an additional significant result of music training on the clustering coefficient of the left mid cingulate gyrus,*F*(1, 67) = 9.527,*p*= 0.003, with a large effect size with a partial*η^2^*of 0.148. Refer to additional results inSupplementary Materials.

**Table 6.** Effects of musical training, active engagement, and emotions on local network metrics.

Brain region	Graph metric	Measure	*df*	*F* value	*p* value	Partial * η ^2^*	Effect size	Survives Bonferroni correction
Left Mid Cingulate Gyrus	Clustering Coefficient	Active Engagement	1, 57	7.707	0.007	0.119	Moderate	No
Left Posterior Cingulate Gyrus	Clustering Coefficient	Active Engagement	1, 57	4.156	0.046	0.068	Small-Moderate	No
Left Hippocampus	Clustering Coefficient	Active Engagement	1, 57	5.349	0.024	0.086	Moderate	No
Left Mid Cingulate Gyrus	Clustering Coefficient	Musical Training	1, 57	7.910	0.007	0.122	Moderate	No
Right Posterior Cingulate Gyrus	Clustering Coefficient	Musical Training	1, 57	6.936	0.011	0.108	Moderate	No
Right Hippocampus	Clustering Coefficient	Musical Training	1, 57	11.389	**0.001**	0.167	Large	**Yes**
Right Thalamus	Clustering Coefficient	Musical Training	1, 57	4.859	0.032	0.079	Moderate	No
Right Mid Cingulate Gyrus	Clustering Coefficient	Emotions	1, 57	5.149	0.027	0.083	Moderate	No
Left Parahippocampal Gyrus	Clustering Coefficient	Emotions	1, 57	7.784	0.007	0.120	Moderate	No
Left Thalamus	Clustering Coefficient	Emotions	1, 57	4.995	0.029	0.081	Moderate	No
Right Hippocampus	Eigenvector Centrality	Active Engagement	1, 57	9.179	**0.004**	0.139	Moderate-Large	**Yes**
Right Hippocampus	Eigenvector Centrality	Musical Training	1, 57	6.459	0.014	0.102	Moderate	No
Left Posterior Cingulate Gyrus	Betweenness Centrality	Active Engagement	1, 57	4.804	0.032	0.078	Moderate	No
Left Anterior Cingulate Gyrus	Node Degree	Musical Training	1, 57	5.367	0.024	0.086	Moderate	No
Right Mid Cingulate Gyrus	Node Degree	Musical Training	1, 57	8.939	**0.004**	0.136	Moderate-Large	**Yes**

Table 6presents ANCOVA findings on the influence of music training, active engagement, and emotions on network metrics across selected brain regions. Data include the targeted brain region, specific network metric, measure type, degrees of freedom (*df*),*F*value,*p*value, partial eta squared (*η²*) for effect size, and the status of Bonferroni correction survivability. Bold refers to findings that survived bonferroni correction.

### 3.5 Impact of musical sophistication and music perception abilities on global network topology

ANCOVAs, with SOMI stage and gender as fixed factors, were conducted to assess the influence of musical measures on global graph metrics, namely global efficiency, characteristic path length, and node degree. Age and years of education were included as covariates to control for their potential influence. The analysis first assessed the influence of the Gold-MSI, followed by the mini-PROMS, and FTT.

The ANCOVA results demonstrated a significant influence of musical training on global efficiency, evidenced by an*F*(1, 57) = 4.612,*p*= 0.036, with a partial*η^2^*of 0.075 suggesting a moderate effect size. This indicates a moderate effect size, suggesting that enhanced musical training is associated with increased efficiency in the global integration of the brain’s neural network. Similarly, musical training exhibited a significant impact on the characteristic path length, with*F*(1, 57) = 4.754,*p*= 0.033, with a partial*η^2^*of 0.077, also indicating a moderate effect size.

The ANCOVA for the mini-PROMS measure revealed a significant effect of melody on node density,*F*(1, 67) = 4.146,*p*= 0.046, but the size of this effect was small (with a partial*η^2^*of 0.058). No significant effects were observed for the FTT measure across global metrics. Refer toFigure 4to see the regional network effects across SOMI stages.

**Fig. 4.**
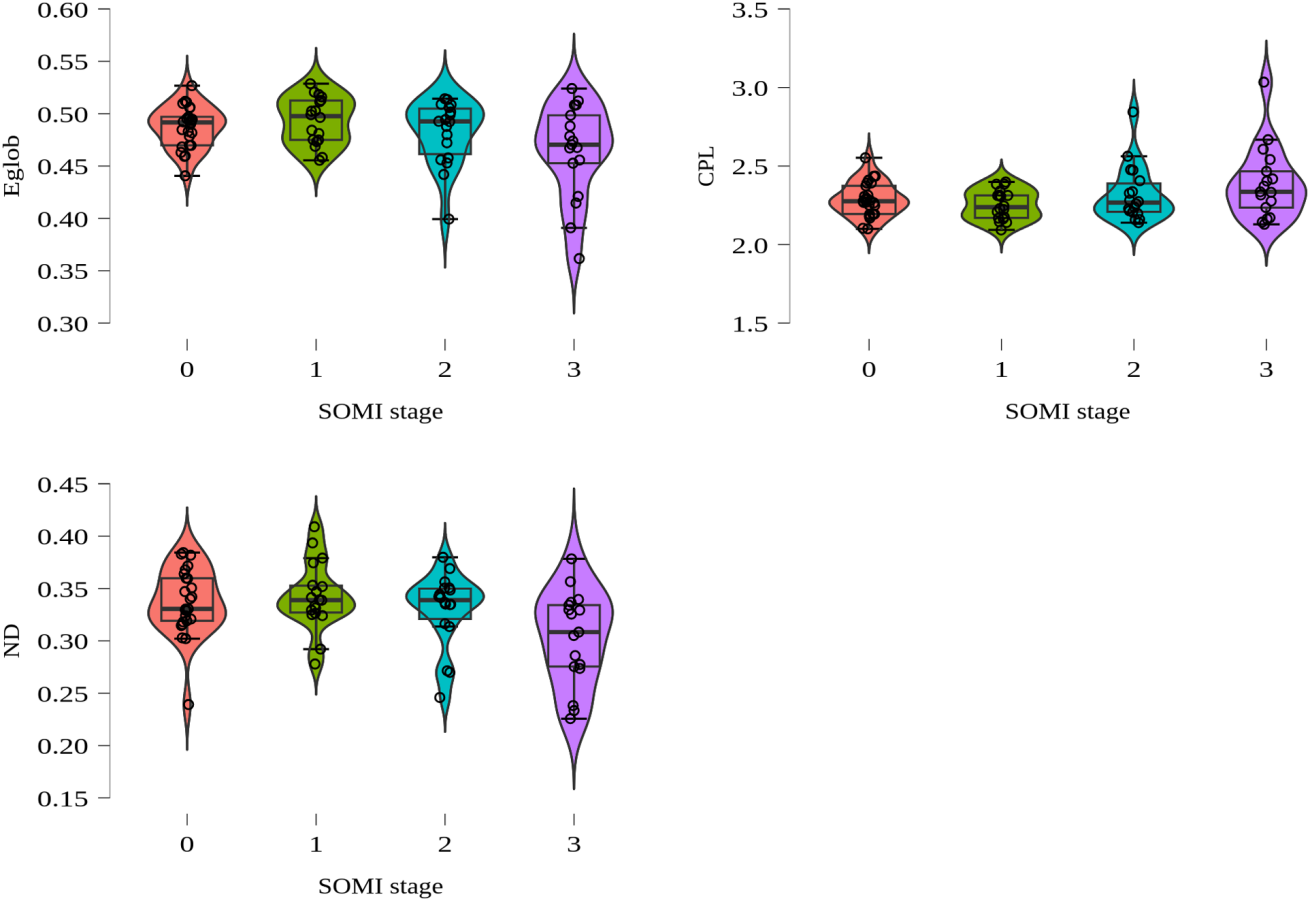
Regional network effects across SOMI stages. Displayed are violin plots showing the regional changes across SOMI stages with regards to graph theoretical metrics; Eglob (Global efficiency), CPL (Characteristic Path Length), and ND (Node Density).

Controlling for diagnosis and gender as fixed factors, and age and years of education as covariates, ANCOVA results demonstrated a significant influence of musical training on global efficiency,*F*(1, 57) = 4.618,*p*= 0.036, with a partial*η^2^*of 0.077, characteristic path length,*F*(1, 57) = 4.350,*p*= 0.042, with a partial*η^2^*of 0.073, and node density,*F*(1, 57) = 4.455,*p*= 0.039, with a partial*η^2^*of 0.075.

The ANCOVA for the mini-PROMS, controlling for diagnosis and gender as fixed factors, and age and years of education as covariates, revealed a significant effect of melody on global efficiency,*F*(1, 67) = 4.361,*p*= 0.041, with a partial*η^2^*of 0.063, and node density,*F*(1, 67) = 6.660,*p*= 0.012, with a partial*η^2^*of 0.093. No significant effects were observed for the FTT measure across global metrics.

## 4 Discussion

This study represents, to our current understanding, a pioneering effort that integrates DTI-based structural connectivity with graph theoretical analysis to explore the influence of musical engagement and music perception abilities on network topology in participants with memory decline. Uniquely, it employs the SOMI framework to assess individuals along the Alzheimer’s Disease continuum, marking the first such endeavor in the field. This innovative approach allows for an examination of the potential neuroprotective effects of music across varying stages of cognitive decline. Moreover, our findings support the evidence indicating music’s capacity in enhancing structural plasticity offering novel insights into the potential protective mechanisms of music and use of music-based interventions against neurodegeneration.

### 4.1 Music perception skills and musical sophistication across SOMI

Firstly, we sought to investigate the impact of music perception skills across different SOMI stages and to examine the protective effects of brain reserve on these abilities in individuals with varying degrees of memory impairment. Contrary to our initial hypothesis, we found no significant differences in music perception skills, encompassing beat, tempo, melody, and tuning perception skills, across the SOMI stages. Further, our analysis found no significant correlations between music perception skills and HBBR, or between music perception skills and the DTI metrics FA and MD of the left and right hippocampus.

The uniformity in music perception skills across different SOMI stages suggests that these abilities might be preserved even in the face of cognitive decline. This preservation could imply that the neural networks underlying music perception are resilient to the neurodegenerative processes associated with memory impairment, and act somewhat independently from other networks known to degenerate in AD. Music, with its rich and complex structure, engages a wide array of cognitive processes, including attention, memory, executive functions, and emotional regulation. The consistent engagement of these widespread neural networks might contribute to the robustness of music perception skills in individuals with or at risk of developing AD.

The absence of a significant correlation between music perception skills and HBBR further complicates our understanding of the protective effects of brain reserve. Traditionally, brain reserve has been conceptualized as the brain’s ability to tolerate pathological changes while maintaining cognitive function, with higher reserve linked to a lower risk of cognitive decline. The lack of correlation observed in this study may suggest that music perception skills are not directly influenced by the structural and functional aspects typically associated with brain reserve, such as brain volume, synaptic count, or neural efficiency. Instead, these skills might be maintained through other mechanisms, possibly related to the unique engagement of the brain by music. Moreover, the lack of correlation between the indices of microstructural change, namely FA and MD, of the left and right hippocampus and music perception skills, further showcases that music perception skills are preserved, even with progressive cognitive decline.

### 4.2 Music perception skills as measured by the mini-PROMS

Next, we investigated the impact of music perception on structural network topological features. We found that the scores for the melody subscale and the total mini-PROMs significantly impacted structural graph metrics across the right hippocampus and the right posterior cingulate cortex (PCC). More broadly, music perception ability also refers to the cognitive and neural processes involved in decoding and understanding complex musical components. This perceptual ability can vary among individuals and is often influenced by prior musical training, exposure, or innate talent. Thus far, one study investigated how individual differences in music perception ability are related to whole-brain white matter structural connectivity (Rajan et al., 2021). The authors found distinct structural network connectivity across fronto-temporal, cerebellar, and cerebro-subcortical regions associated with music processing abilities. Further, they found increased modularity in brain networks associated with higher music perception abilities with the right PCC emerging as a significant hub within the networks.

In line with their findings of the right PCC, our results demonstrated that the melody subscale of the mini-PROMS significantly influenced the C_i_in both the right PCC and right hippocampus, with notably large effect sizes. The PCC is a node of the brain’s default mode network (DMN;Buckner et al., 2008), a prominent intrinsic brain network involved in various aspects of autobiographical memory, self-referential thought, and emotion processing. The DMN has also been known to be compromised in the progression of AD. This is due to its spatial overlap with patterns of AD-related atrophy and tauopathy, making it particularly vulnerable to AD pathology (Hampton et al., 2020). Additionally, decreased DMN connectivity has been documented in participants with mild or preclinical AD, particularly in the PCC and precuneus (Simic et al., 2014). This suggests that the breakdown of this essential network may occur in the very early stages of AD (Xie et al., 2019).

The PCC has been shown to be engaged when recalling a musical piece of personal significance (Janata, 2009). This includes the DMN showing that familiar music engages this network more strongly than unfamiliar music (Castro et al., 2020). Moreover, the PCC plays a role in internally directed thought, which contributes to the introspective and creative aspects of music. Conversely, the right hippocampus, which is crucial for memory formation and has been linked to emotional processing and music-evoked emotions (Koelsch, 2014,^2020^), has been shown to be involved in encoding and retrieving musical memories. This includes melodies, lyrics, and contextual details in which a song was first heard and thus encoded (Watanabe et al., 2008). Therefore, the recognition aspect involved in familiar melodies also connects to the autobiographical memory. Furthermore, the findings indicate that melodic perception not only stimulates activity in the right hippocampus but also enhances its functional integration within the brain’s memory networks. As this area is associated with linking musical elements with autobiographical memories and emotions, this reveals the therapeutic role of melody in evoking vivid and emotionally salient memories. Thus, the effects on the C_i_reveal a more densely networked group of regions, suggesting higher interconnection. These changes may be attributed to structural and functional changes due to musical training, as observed by other research (Olszewska et al., 2021). The structural connectivity of the right hippocampus, therefore, plays a crucial role in supporting the cognitive and emotional aspects of musical engagement, suggesting that enhancing melodic perception could contribute to cognitive resilience and emotional health.

Beat perception is a fundamental aspect of music cognition that involves the ability to perceive a regular pulse or beat in musical rhythms. Interestingly, our findings suggest a link between beat perception and increased betweenness centrality in the right hippocampus. This centrality reflects the hippocampus’s influence in integrating information across brain regions. By facilitating the encoding and retrieval of rhythmic patterns, beat perception could strengthen connections between the hippocampus and brain regions involved in auditory processing, potentially influencing memory formation and recall specific to rhythmic information. The hippocampus plays a role in processing, predicting, and storing rhythmic information, which could be a key neural mechanism underlying our ability to perceive and synchronize with rhythm (Billig et al., 2022). Further, rhythm also plays a crucial role in social interactions and communication. Our ability to perceive and synchronize with rhythm might have facilitated social cohesion and cooperation within early human groups (Koelsch, 2011). However, the relationship between beat perception and brain structure is complex and not fully understood, necessitating further research to clarify the neural mechanisms and its implications for cognition and behavior.

Overall, our findings reveal the significant potential for developing music-based therapeutic interventions for individuals with worsening memory impairments, as music perception, musical training, and active engagement seem to be associated with increased structural connectivity of key regions involved in the processing of emotion, memory, and learning processes. This suggests that offering a non-invasive approach can enhance cognitive resilience in aging populations, specifically those at risk of developing AD.

### 4.3 Musical behaviors as assessed via the Gold-MSI

In our study, we used the Gold-MSI to assess and quantify musical sophistication among our participants (Müllensiefen et al., 2014). This questionnaire evaluates various aspects of proficiency and engagement in music. We selected the active engagement and musical training subscales, in addition to selected questions from the emotions subscale evaluating these responses with structural connectivity metrics. Globally, our findings indicate that musical training appears to influence two key graph parameters, global efficiency and characteristic path length. Global efficiency is a measure of how efficiently a network exchanges information, and can be further defined as the average efficiency over all pairs of nodes in the network (Ek et al., 2015). This provides an overview of the overall capacity for parallelizing information transfer and integration among distributed components of the system. Characteristic path length defines the average number of steps along the shortest paths for all possible pairs of nodes in a network (Fornito et al., 2013), as it measures the efficiency of information along a network. The shorter the average path length, the quicker and more efficient information can transfer across the network, thus further defining it as a measure of a network’s interconnectedness (Van Den Heuvel et al., 2009). The moderate effect sizes observed for both global efficiency and characteristic path length suggest a significant neural impact of musical training. Musical training may play a role in optimizing the efficiency and interconnectedness of structural brain networks, potentially reflecting enhanced cognitive processing capabilities in individuals with higher musical training. Although the sample size is limited, the findings suggest that musical training in one’s life time has significant effects on regional efficiency adding to cognitive resilience.

Locally, our examination of the clustering coefficient, C_i_, a graph metric that quantifies the degree to which nodes in a graph tend to cluster together, provides insights into the organization and local interconnectivity within neural networks. Our findings revealed that musical training significantly influenced the C_i_of the right hippocampus, an area critical for the processing of spatial navigation and contextual information. This suggests that musical training enhances not only the connectivity but also the integration of neural networks in this region. The right hippocampus is integral to various aspects of musical processing, including memory, emotional response, and processing (Frühholz et al., 2014;Koelsch, 2020;Immordino-Yang & Singh, 2013), and recollection and familiarity recognition (Watanabe et al., 2008;Wixted & Squire, 2010). Additionally, it appears to play a crucial role in musical memory, which is relatively spared in individuals with AD (Groussard et al., 2019;Jacobsen, Stelzer, Fritz, Chételat, et al., 2015). This highlights the potential of music therapy for individuals with memory impairments, as its application may lead to enhanced cognitive buffering while offering potential rehabilitative capabilities against worsening cognitive decline.

Supporting our observations, studies have demonstrated increased functional plasticity in the hippocampi of musicians compared to non-musicians (Herdener et al., 2010). This is further corroborated by research indicating both structural and functional changes as a result of long-term musical training, leading to cognitive differences between musicians and non-musicians (Gaser & Schlaug, 2003;Groussard et al., 2010;Li et al., 2018). These changes are particularly pronounced in individuals who begin training before the age of seven, showcasing the sensitivity of the developing brain to musical exposure (Hyde et al., 2009).

For the understanding of the effects of musical engagement on a possible deceleration, or perhaps even amelioration, of cognitive decline, it is interesting to consider the concept of adult neurogenesis (Kempermann et al., 2015), that is, the generation of new neurons in the brain, particularly in areas like the subventricular zone and the subgranual zone of the dentate gyrus of the hippocampal formation. Meta-analytic data indicate that music-evoked emotions are associated with activity changes in the anterior hippocampal formation, leading to the possibility that emotions evoked with music also stimulate neurogeneration, thus having beneficial effects against cognitive decline (Koelsch, 2020). Moreover, musical engagement is a complex sensory-motor activity, and has been linked to increased structural white matter integrity, particularly in lifelong musicians, suggesting that engaging with such musical activities across the lifespan can either decelerate or reverse white matter atrophy commonly associated with healthy aging (Andrews et al., 2021). These findings underscore the brain’s ability to build resilience and adapt over the life course through engaging in musical activities.

Furthermore, the active engagement subscale of the Gold-MSI influenced the eigenvector centrality of the right hippocampus. Active engagement measures the extent of current involvement in music-related activities. Eigenvector centrality is a graph theoretical measure of the influence of a node in a network. EC is based on the principles that a node’s importance is enhanced if it connected to other important nodes, that is, a node with higher EC reveals a greater interconnectivity between nodes that also have high EC. The significant influence of active engagement on the EC of the right hippocampus reveals that active engagement alone can alter interconnectivity. Although the effect was moderate, this relates to research findings that receptive music listening therapy can positively impact individuals with AD, offering them access to their autobiographical memories, specifically when the music they listen to is familiar (Cuddy & Duffin, 2005;Cuddy et al., 2015;El Haj et al., 2012). Daily active engagement through the life course may strengthen connectivity in the hippocampus, enhancing the retrieval of autobiographical memories, even as memory decline increases.

Interestingly, research using the Gold-MSI has shown that musical training enhances auditory-motor cortex coupling, which is crucial for music and speech perception (Rimmele et al., 2022). This enhanced coupling reveals that the skills and perceptual abilities honed through musical training extend to broader auditory-motor integration, which is a crucial component of cognitive and neural functioning. Additionally, the predictive power of the Gold-MSI, specifically regarding musical training and perceptual abilities, showcases the significant influence of musical sophistication on speech auditory-motor synchronization strength. Individuals who engage more intensely with music showed an increased capacity for auditory-motor integration compared to those with lower levels of musical engagement. We did not consider using the perception abilities subscale in our paper, however we did utilize the mini-PROMS to gauge music perception ability. Nevertheless, we found that active musical engagement had an influence on structural network connectivity in the right hippocampus.

### 4.4 Limitations

This study explores the impact of music on brain connectivity related to memory and emotional processing in individuals with or at risk of developing AD, noting several limitations. The main limitation in our study is the lack of physiological biomarkers. Individuals with AD or MCI who were accepted as part of our study had a diagnosis from a physician, but individuals with probable MCI, SCD, or sub-threshold SCD were not verified for existing biomarkers. Furthermore, factors like the type and extent of musical training, starting age, and individual abilities could significantly affect the outcomes. Although findings suggest a possible connection between musical activity and brain connectivity, a causal link cannot be established, and further experimental and longitudinal studies are required.

Further methodological limitations include our small sample size and cross-sectional implementation, which, along with reliance on diffusion MRI, can lack in resolution and specificity, restricts the ability to conclusively determine effects, and calls for advanced neuroimaging and analytical methods in future work. Our choices may have yielded results that were not sensitive enough to detect subtle variations in brain structure related to musical training.

Additionally, the analysis could have benefited from additional b-zero values and higher b-values to increase resolution. Insufficient encoding directions may lead to a significant risk of oversimplifying complex fiber configurations, and lead to inaccuracies in finding crossing fibers and other intricate white matter structures. To address these limitations and improve the accuracy of fiber tractography, future studies should employ advanced models like spherical deconvolution, which may better capture the complexities of white matter microstructure. Lastly, focusing on individuals with memory decline limits the generalizability of the results to wider populations with different cognitive conditions or healthy subjects, indicating a vast area for extended research.

## 5 Conclusion

Our paper introduces several novel aspects to the field of neurodegenerative research, particularly regarding the use of music for memory decline. The study employs an innovative methodological approach by combining graph theoretical methods with diffusion MRI to examine the impact of music on the brain’s structural networks. Our investigation highlights how musical behaviors and musical perception skills significantly impact structural network connectivity, focusing specifically on regions within the Papez circuit, a crucial limbic circuit related to memory and emotional processing. The study uniquely integrates these musical measures with SOMI staging, allowing for a nuanced exploration of how musical abilities and active engagement might influence structural network topology across different stages of memory impairment.

Our findings reveal that musical perception skills, such as the ability to perceive melody and beat, can modulate neurodegeneration in regions known to be affected in AD, particularly within the Papez circuit, including areas such as the right hippocampus and right posterior cingulate cortex. Musical training impacts topological metrics of the right hippocampus and right mid cingulate gyrus, while active musical engagement influences the eigenvector centrality of the right hippocampus. Furthermore, musical training was shown to impact global efficiency and characteristic path length, highlighting its role in shaping network topology. This integration is particularly relevant for understanding and potentially managing AD, offering new insights into the role of non-pharmacological therapies like music therapy in bolstering brain networks and potentially mitigating the effects of neurodegeneration and cognitive decline in individuals with or at risk of developing AD.

## Supplementary Material

Supplementary Material

## Data Availability

The datasets generated and/or analyzed during the current study are not publicly available. However, datasets and code are available from the corresponding author upon reasonable request. Requests for access to these data should be addressed to Anna Maria Matziorinis or Stefan Koelsch, who will consider such requests in accordance with the ethical guidelines and legal requirements pertinent to the data in question.
