# Supplementary material for: The effects of musicality on brain network topology in the context of Alzheimer’s disease and memory decline
Source: Imaging Neurosci (Camb). 2024 Aug 5;2:imag-2-00248. doi: 10.1162/imag_a_00248 (PMC12272188; doi:10.1162/imag_a_00248)
Supplement: Supplementary Material [file imag_a_00248-supp.pdf]

Supplementary Materials

1. Methods

1.1 Descriptives

Table 2 Descriptive statistics and demographics across SOMI stages (*N* = 78)

	**SOMI-0**	**SOMI-1**	**SOMI-2**	**SOMI-3**	**SOMI-4**
n	n = 25	n = 18	n = 12	n = 6	n = 17
age	65.32 (10.72)	69.56 (10.42)	74.50 (4.93)	78.00 (6.42)	76.24 (6.34)
gender (m/f)	9/16	9/9	6/6	2/4	9/8
years of education	14.92 (3.29)	13.67 (2.72)	13.83 (3.10)	11.33 (3.56)	14.71 (3.24)
handedness (right)	25	18	12	6	17
medication type (% yes)					
Alzheimer's	0.0	0.0	0.0	0.0	29.4
sleep	4.0	5.6	8.3	0.0	0.0
allergy	16.0	22.2	0.0	16.7	0.0
blood pressure	28.0	50.0	41.7	50.0	41.2
metabolism	12.0	5.6	8.3	16.7	11.8
anxiety	0.0	0.0	0.0	16.7	0.0
antidepressants	0.0	0.0	16.7	0.0	0.0
cholesterol	12.0	27.8	16.7	16.7	29.4
other	36.0	61.1	50.0	0.0	29.4
smoking (% yes)	4.00	5.6	0.0	0.0	5.9
smoking (previously)	24.0	22.2	16.7	0.0	17.6

Data was divided into SOMI stages: SOMI-0 through SOMI-4. Listed values included sample size (n), mean age and standard deviation, gender (sex at birth), either Male or Female, mean years of education, and standard deviation. Handedness is listed as those who are right-handed. Medication type listed in terms of affirmative use of medication type (percentage of those who replied yes to using medication).

Table 2 Descriptive statistics and demographics across SOMI stages (*N* = 78)

	**SOMI-0**	**SOMI-1**	**SOMI-2/3**	**SOMI-4**
n	n = 25	n = 18	n = 18	n = 17
age	65.32 (10.72)	69.56 (10.42)	75.67 (5.54)	76.24 (6.34)
gender (m/f)	9/16	9/9	8/10	9/8
years of education	14.92 (3.29)	13.67 (2.72)	13.00 (3.38)	14.71 (3.24)
handedness (right)	25	18	17	17
medication type (% yes)				
Alzheimer's	0.0	0.0	0.0	29.4
sleep	4.0	5.6	5.6	0.0
allergy	16.0	22.2	5.6	0.0
blood pressure	28.0	50.0	44.4	41.2
metabolism	12.0	5.6	11.1	11.8
anxiety	0.0	0.0	5.6	0.0

antidepressants	0.0	0.0	11.1	0.0
cholesterol	12.0	27.8	16.7	29.4
other	36.0	61.1	33.3	29.4
smoking (% yes)	4.00	5.6	0.0	5.9
smoking (previously)	24.0	22.2	11.1	17.6

Data was divided into SOMI stages: SOMI-0 through SOMI-4. Listed values included sample size (n), mean age and standard deviation, gender (sex at birth), either Male or Female, mean years of education, and standard deviation. Handedness is listed as those who are right-handed. Medication type listed in terms of affirmative use of medication type (percentage of those who replied yes to using medication).

Table 3 Cognitive, Memory, Music measure scores, and Hippocampal-Based Brain Reserve across SOMI stages (*N* = 78)

	**SOMI-0**	**SOMI-1**	**SOMI-2**	**SOMI-3**	**SOMI-4***
Sample size	n = 25	n = 18	n = 12	n = 6	n = 17
MMSE total	28.44 (1.69)	28.11 (2.06)	27.92 (1.44)	25.33 (2.42)	22.12 (4.05)
pFCSRT-IR					
total free recall	34.16 (2.66)	27.17 (1.82)	22.00 (1.48)	17.50 (5.09)	9.17 (9.36)
total recall	47.92 (0.28)	47.72 (0.46)	47.83 (0.39)	45.83 (0.41)	28.25 (16.64)
delayed free recall	12.72 (1.49)	10.94 (1.47)	9.17 (2.29)	6.83 (1.47)	3.42 (4.34)
delayed cued recall	3.20 (1.53)	5.00 (1.46)	6.83 (2.29)	8.67 (1.51)	6.25 (3.79)
Gold-MSI					
active engagement	3.18 (1.24)	3.35 (0.99)	3.76 (0.91)	4.40 (0.91)	3.21 (1.30)
musical training	2.19 (1.24)	2.46 (1.19)	2.60 (1.27)	2.51 (1.20)	2.37 (1.03)
emotions	4.70 (1.49)	4.84 (1.20)	4.76 (1.34)	5.20 (1.54)	4.47 (1.34)
mini-PROMS					
melody	5.22 (1.60)	5.25 (1.66)	5.63 (1.21)	4.75 (2.42)	4.59 (1.95)
tuning	4.32 (1.42)	4.48 (1.62)	4.08 (1.14)	4.08 (1.59)	3.91 (0.89)
beat	4.80 (1.54)	4.22 (1.33)	4.54 (1.30)	4.33 (1.08)	4.71 (1.48)
tempo	5.12 (1.94)	5.39 (1.48)	5.58 (1.28)	4.25 (2.09)	4.44 (1.43)
PROMS total score	19.46 (4.28)	19.44 (4.95)	19.83 (3.18)	17.42 (5.23)	17.65 (4.53)
Finger-tapping task (FTT)					
left-hand	48.36 (7.68)	45.89 (7.32)	45.69 (7.34)	42.71 (6.04)	39.00 (11.83)
right-hand	52.81 (7.38)	48.66 (7.05)	48.54 (6.73)	38.17 (6.25)	43.41 (16.16)
combined average	50.58 (6.95)	47.28 (6.56)	47.11 (6.31)	40.44 (4.92)	41.21 (13.76)
HBBR	0.29 (0.74)	0.43 (0.68)	-0.13 (0.69)	-0.05 (1.33)	-0.58 (0.77)

Abbreviations: Mini-mental state examination (MMSE); Goldsmiths sophistication index (Gold-MSI); Mini-version of the profile of music perception skills (mini-PROMS) including four subscales; melody, tuning, beat, tempo, and Proms total score; finger-tapping task (FTT), including left-hand, right-hand, and combined average score, and Hippocampal-based brain reserve *z* score*s* (HBBR). The listed values are means and standard deviations.
* = missing data.

Table 3 Music measure scores across SOMI stages (*N* = 78)

	**SOMI-0**	**SOMI-1**	**SOMI-2/3**	**SOMI-4***
Sample size	n = 25	n = 18	n = 18	n = 17

MMSE total	28.44 (1.69)	28.11 (2.06)	27.05 (2.16)	22.12 (4.05)
pFCSRT-IR				
total free recall	34.16 (2.66)	27.17 (1.82)	20.50 (3.71)	9.17 (9.36)
total recall	47.92 (0.28)	47.72 (0.46)	47.17 (1.04)	28.25 (16.64)
delayed free recall	12.72 (1.49)	10.94 (1.47)	8.39 (2.30)	3.42 (4.34)
delayed cued recall	3.20 (1.53)	5.00 (1.46)	7.44 (2.20)	6.25 (3.79)
Gold-MSI				
active engagement	3.18 (1.24)	3.35 (0.99)	3.96 (1.04)	3.21 (1.30)
musical training	2.19 (1.24)	2.46 (1.19)	2.57 (1.21)	2.37 (1.03)
emotions	4.70 (1.49)	4.84 (1.20)	4.90 (1.37)	4.47 (1.34)
mini-PROMS				
melody	5.22 (1.60)	5.25 (1.66)	5.33 (1.69)	4.59 (1.95)
tuning	4.32 (1.42)	4.48 (1.62)	4.08 (1.26)	3.91 (0.89)
beat	4.80 (1.54)	4.22 (1.33)	4.47 (1.21)	4.71 (1.48)
tempo	5.12 (1.94)	5.39 (1.48)	5.14 (1.66)	4.44 (1.43)
PROMS total score	19.46 (4.28)	19.44 (4.95)	19.03 (3.99)	17.65 (4.53)
Finger-tapping task (FTT)				
left-hand	48.36 (7.68)	45.89 (7.32)	44.81 (6.94)	39.00 (11.83)
right-hand	52.81 (7.38)	48.66 (7.05)	45.49 (8.04)	43.41 (16.16)
combined average	50.58 (6.95)	47.28 (6.56)	45.15 (6.58)	41.21(13.76)
HBBR	0.29 (0.74)	0.43 (0.68)	-0.10 (0.91)	-0.58 (0.77)

Abbreviations: Mini-mental state examination (MMSE); Goldsmiths sophistication index (Gold-MSI); Mini-version of the profile of music perception skills (mini-PROMS) including four subscales; melody, tuning, beat, tempo, and Proms total score; finger-tapping task (FTT), including left-hand, right-hand, and combined average score, and Hippocampal-based brain reserve *z* scores (HBBR). The listed values are means and standard deviations.
* = missing data.

1.2 Goldsmith-Musical Sophistical Index, selected questions, 19 in total used for study

Sample question #1: 'I spend a lot of my free time doing music-related activities.'

Reversed negative scored items: 5, 6, 9 and 11.

Gold-MSI Scorer: https://shiny.gold-msi.org/gmsiscorer/

Questions about your musical background

**Please circle the most appropriate category:**	**1 Comple tely dis-agree**	**2 Strongl y dis-agree**	**3 Dis-agree**	**4 Neither agree nor dis-agree**	**5 Agre e**	**6 Strong ly agree**	**7 Comple tely agree**
1. I spend a lot of my free time doing	**1**	**2**	**3**	**4**	**5**	**6**	**7**

music-related activities.							
2. I sometimes choose music that can trigger shivers down my spine.	**1**	**2**	**3**	**4**	**5**	**6**	**7**
3. I enjoy writing about music, for example on blogs and forums.	**1**	**2**	**3**	**4**	**5**	**6**	**7**
4. I'm intrigued by musical styles I'm not familiar with and want to find out more.	**1**	**2**	**3**	**4**	**5**	**6**	**7**
5. Pieces of music rarely evoke emotions for me.	**1**	**2**	**3**	**4**	**5**	**6**	**7**
6. I have never been complimented for my talents as a musical performer.	**1**	**2**	**3**	**4**	**5**	**6**	**7**
7. I often read or search the internet for things related to music.	**1**	**2**	**3**	**4**	**5**	**6**	**7**
8. I often pick certain music to motivate or excite me.	**1**	**2**	**3**	**4**	**5**	**6**	**7**
9. I don't spend much of my disposable income on music.	**1**	**2**	**3**	**4**	**5**	**6**	**7**
10. Music is kind of an addiction for me - I couldn't live without it.	**1**	**2**	**3**	**4**	**5**	**6**	**7**
11. I would not consider myself a musician.	**1**	**2**	**3**	**4**	**5**	**6**	**7**
12. I keep track of new music that I come across (e.g. new artists or recordings).	**1**	**2**	**3**	**4**	**5**	**6**	**7**
13. I engaged in regular, daily practice of a musical instrument (including voice) for … years.	**0**	**1**	**2**	**3**	**4-5**	**6-9**	**> 9**
14. At the peak of my interest, I practiced … hours per day on my primary instrument.	**0**	**0,5**	**1**	**1,5**	**2**	**3-4**	**> 4**
15. I have attended ... live music events as an audience member in the past twelve months.	**0**	**1**	**2**	**3**	**4-6**	**7-10**	**> 10**
16. I have had formal training in music theory for … years.	**0**	**0,5**	**1**	**2**	**3**	**4-6**	**> 6**
17. I have had … years of formal training on a musical instrument (including voice) during my lifetime.	**0**	**0,5**	**1**	**2**	**3-5**	**6-9**	**> 9**
18. I can play ... musical instruments.	**0**	**1**	**2**	**3**	**4**	**5**	**> 5**
19. I listen attentively to music for … per day.	**0-15 min**	**15-30 min**	**30-60 min**	**60-90 min**	**2 hrs**	**2-3 hrs**	**> 3 hrs**

Questions and Subscales:

(1) Active Engagement (9 total questions): 1, 3, 4, 7, 9, 10, 12, 15, 19
(2) Musical Training (7 total questions): 6, 11, 13, 14, 16, 17, 18
(3) Emotions (3 total questions): 2, 5, 8

1.3 Sample of Preprocessed Diffusion Image, Tractography, and Connectivity Matrices

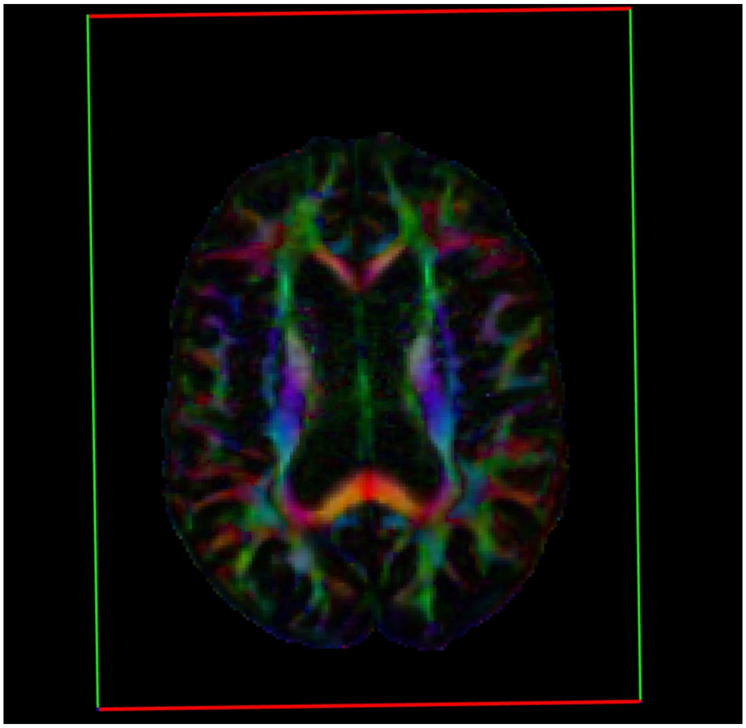

Figure 1. Sample pre-processed DTI corrected image for one participant

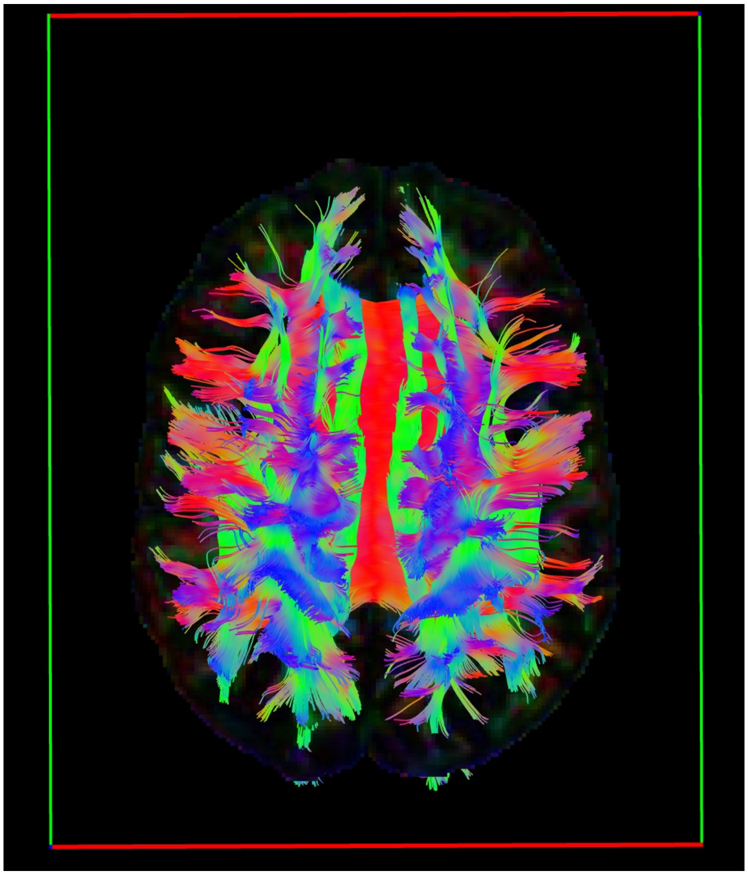

Figure 2. Sample whole-brain tractogram for one participant

‘

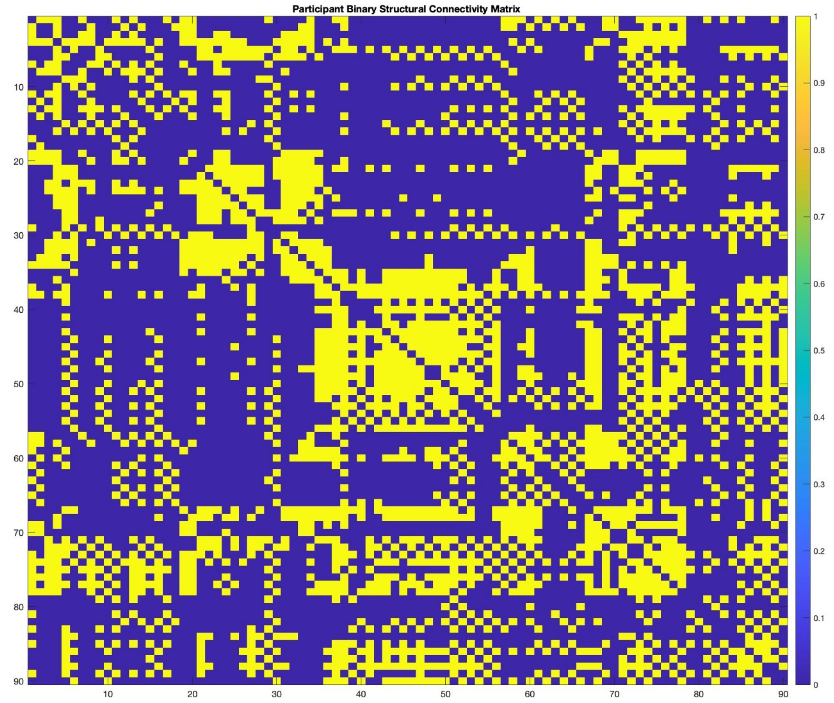

Figure 3. Sample binary structural connectivity matrix with self-connections set to 0 for one participant

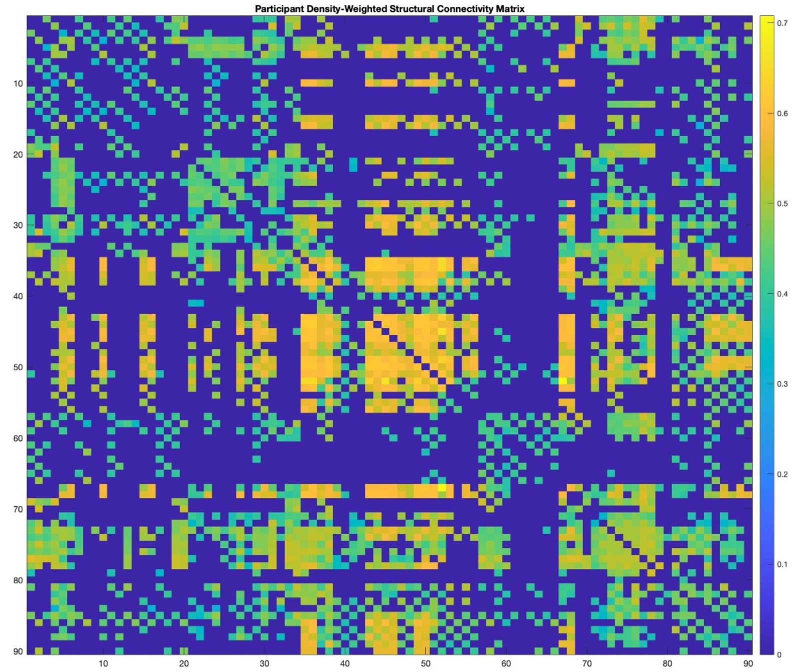

Figure 4. Sample density weighted structural connectivity matrix with self-connections set to 0 for one participant

1.4 Derivation of DTI-based metrics

1.4.1 Fractional Anisotropy

Fractional Anisotropy (FA) measures the degree of anisotropy of a diffusion process.

$$FA = \sqrt{\frac{3}{2}} \sqrt{\frac{(\lambda_1 - \lambda_{avg})^2 + (\lambda_2 - \lambda_{avg})^2 + (\lambda_3 - \lambda_{avg})^2}{\lambda_1^2 + \lambda_2^2 + \lambda_3^2}}$$

where λ_1, λ_2, and λ_3 are the eigenvalues of the diffusion tensor, and $\lambda_{avg} = \frac{\lambda_1 + \lambda_2 + \lambda_3}{3}$.

1.4.2 Mean Diffusivity

Mean Diffusivity (MD) is the average diffusion coefficient of a tissue and quantifies the rate at which water molecules diffuse within the tissue.

$$MD = \frac{\lambda_1 + \lambda_2 + \lambda_3}{3}$$

where λ_1, λ_2, and λ_3 are the eigenvalues of the diffusion tensor.

1.5 Graph Theoretical Metrics

1.5.1 Equations

Graph theoretical metrics used the Brain Connectivity Toolbox for computation of measures.

Definition of regional graph metrics

I. Nodal Density (D_i) refers to the proportion of actual connections to potential connections for a given node in the graph.

For a node i, the nodal density D_i is given by $D_i = \frac{k_i}{N-1}$

where k_i is the degree of node i (i.e., the number of connections it has), and N is the total number of nodes in the graph.

II. Global Efficiency (E_{glob}) is a measure of the efficiency of information transfer in the network.

It is given by $E_{glob} = \frac{1}{N(N-1)} \frac{\sum_{i \neq j \in G} 1}{d(i,j)}$

where N is the number of nodes, $d(i,j)$ is the shortest path length between nodes i and j.

III. Characteristic Path Length (L) is the average shortest path length in a network.

It is calculated as $L = \frac{1}{N(N-1)} \sum_{i \neq j \in G} d(i,j)$

where $d(i,j)$ is the shortest path length between nodes i and j.

Definition of local graph theoretical metrics

I. Clustering Coefficient (C_i) measures the degree to which nodes in a graph tend to cluster together.

For a node i, the clustering coefficient C_i is $C_i = \frac{2T(i)}{k_i(k_i - 1)}$

where $T(i)$ is the number of triangles through node i and k_i is the degree of i.

II. Eigenvector Centrality measures the influence of a node in a network.

It is calculated as the eigenvector corresponding to the largest eigenvalue of the adjacency matrix A.

III. Betweenness Centrality (C_B) quantifies the number of times a node acts as a bridge along the shortest path between two other nodes.

It is given by $C_B(i) = \frac{\sum_{s \neq i \neq t \in G} \sigma_{st}(i)}{\sigma_{st}}$

where σ_{st} is the total number of shortest paths from node s to node t and $\sigma_{st}(i)$ is the number of those paths passing through i.

IV. Nodal Degree (k_i) is the number of connections a node has.

For a node i, its degree k_i is given by $k_i = \sum_j A_{ij}$

where A is the adjacency matrix, A_{ij} is 1 if there is a connection between nodes i and j, and 0 otherwise.

2. Results

2.1 Mini-PROMS across SOMI stages

An extensive analysis was conducted to explore the relationship between the SOMI and various aspects of music perception. The variables of interest included beat perception, tempo recognition, melody discrimination, tuning sensitivity, and the aggregate score on the mini-PROMS, which collectively aim to provide a comprehensive measure of an individual's music perception capabilities.\

The results indicated no significant differences in music perception skills encompassing beat, tempo, melody, tuning, and the total mini-PROMS score across the SOMI stages. The analyses yielded the following findings:

Beat Perception: No significant variation was observed in beat perception abilities across different SOMI stages, $F(3, 68) = 0.766$, $p = 0.517$. Tempo Recognition: Similarly, tempo recognition did not differ significantly among participants categorized into various stages of memory impairment, $F(3, 68) = 0.675$, $p = 0.570$. Melody Discrimination: Analysis of melody discrimination skills across SOMI stages also showed no significant differences, $F(3, 68) = 0.625$, $p = 0.602$. Tuning Sensitivity: Tuning sensitivity remained consistent across stages of objective memory impairment, with no significant variances detected, $F(9, 68) = 0.887$, $p = 0.541$. Total mini-PROMS Score: The overall music perception ability, as indicated by the total mini-PROMS score, did not significantly change with varying stages of memory impairment, $F(3, 68) = 1.316$, $p = 0.277$.

Table 5. Correlation table of SOMI stages with MMSE and CERAD word learning scores

Correlation Table

		Pearson		Spearman	
		**r**	**p**	**rho**	**p**
SOMI	MMSE Total	-0.617***	1.738×10^{-9}	-0.584***	1.960×10^{-8}
SOMI	Word learning Recognition	-0.697***	1.370×10^{-12}	-0.728***	4.054×10^{-14}
SOMI	Word Learning total	-0.673***	1.518×10^{-11}	-0.674***	1.299×10^{-11}
SOMI	Word learning Delayed Recall	-0.782***	4.605×10^{-17}	-0.762***	8.472×10^{-16}

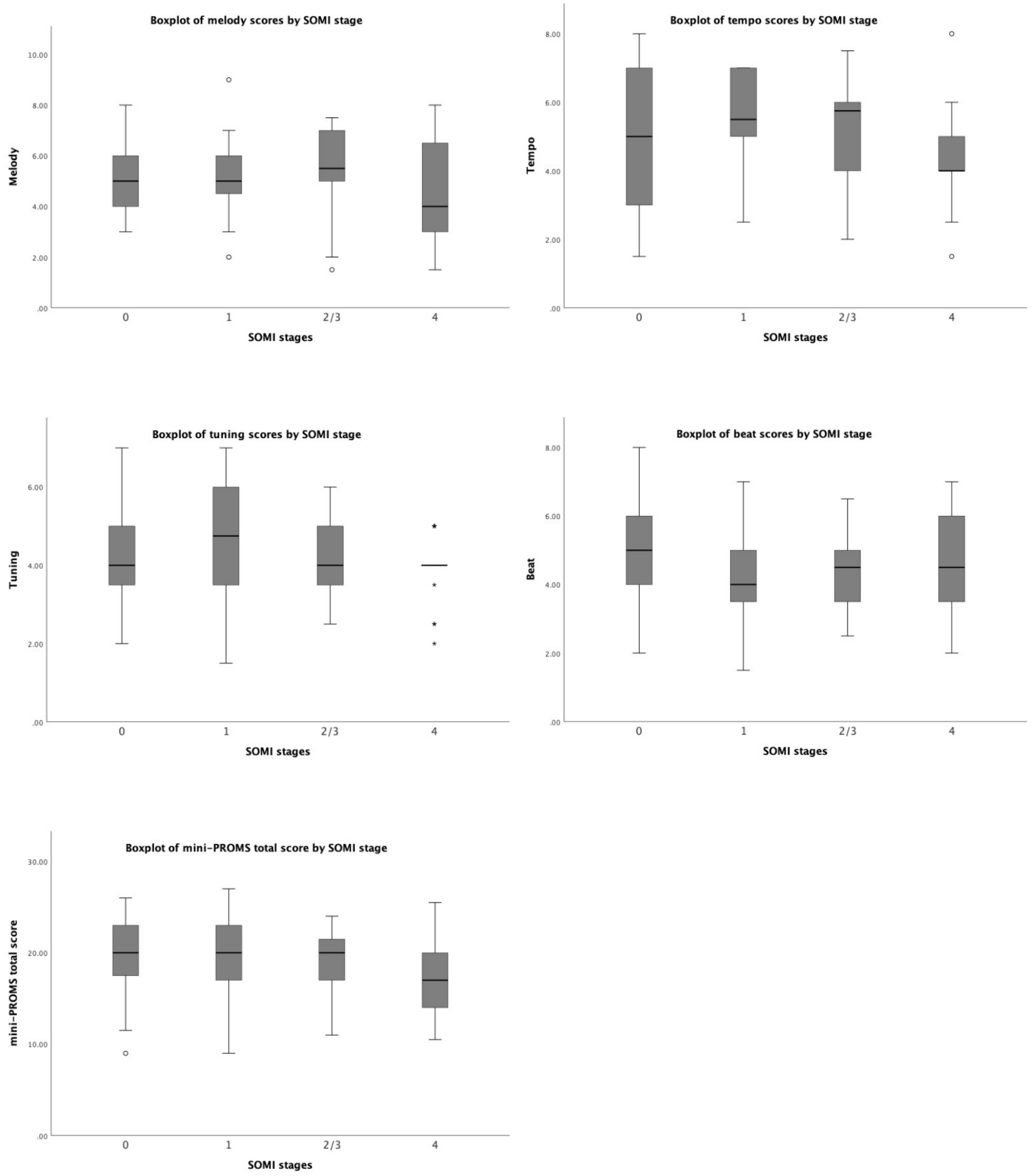

Figure 5. Presents boxplots of the scores for each mini-PROMS subscale, illustrating the distribution of test scores across the SOMI categories.

Table 6 Effects of Musical Training, Active Engagement, and Emotions on Local Network Metrics – Controlling for Diagnosis

Brain Region	Graph Metric	Measure	*df*	*F* Value	*p* Value	Partial η^2	Effect Size	Survives Bonferroni Correction
Left Mid Cingulate Gyrus	Clustering Coefficient	Active Engagement	1, 57	7.252	0.009	0.116	Moderate	No
Left Posterior Cingulate Gyrus	Clustering Coefficient	Active Engagement	1, 57	5.248	0.026	0.087	Moderate	No
Left Hippocampus	Clustering Coefficient	Active Engagement	1, 57	4.866	0.032	0.081	Moderate	No
Right Hippocampus	Clustering Coefficient	Active Engagement	1, 57	4.214	0.045	0.071	Moderate	No
Left Parahippocampal Gyrus	Clustering Coefficient	Active Engagement	1, 57	5.432	0.023	0.090	Moderate	No
Left Mid Cingulate Gyrus	Clustering Coefficient	Musical Training	1, 57	9.527	0.003	0.148	Large	Yes
Right Posterior Cingulate Gyrus	Clustering Coefficient	Musical Training	1, 57	4.139	0.048	0.086	Moderate	No
Right Hippocampus	Clustering Coefficient	Musical Training	1, 57	12.355	<0.001	0.183	Large	Yes
Left Parahippocampal Gyrus	Clustering Coefficient	Musical Training	1, 57	5.307	0.025	0.088	Moderate	No
Right Thalamus	Clustering Coefficient	Musical Training	1, 57	7.456	0.008	0.119	Moderate	No
Left Anterior Cingulate Gyrus	Clustering Coefficient	Emotions	1, 57	4.345	0.042	0.073	Moderate	No
Left Mid Cingulate Gyrus	Clustering Coefficient	Emotions	1, 57	4.821	0.032	0.081	Moderate	No
Left Parahippocampal Gyrus	Clustering Coefficient	Emotions	1, 57	8.673	0.005	0.136	Moderate-Large	No
Left Posterior Cingulate Gyrus	Eigenvector Centrality	Active Engagement	1, 57	4.995	0.039	0.081	Moderate	No
Right Hippocampus	Eigenvector Centrality	Active Engagement	1, 57	9.003	0.004	0.141	Large	Yes
Right Hippocampus	Eigenvector Centrality	Musical Training	1, 57	6.258	0.015	0.102	Moderate	No
Right Thalamus	Eigenvector Centrality	Musical Training	1, 57	5.862	0.019	0.096	Moderate	No
Left Posterior Cingulate Gyrus	Eigenvector Centrality	Emotions	1, 57	4.077	0.048	0.069	Small-Moderate	No
Right Hippocampus	Eigenvector Centrality	Emotions	1, 57	5.144	0.027	0.086	Moderate	No
Left Parahippocampal Gyrus	Betweenness Centrality	Emotions	1, 57	4.431	0.040	0.075	Moderate	No
Left Anterior Cingulate Gyrus	Node Degree	Musical Training	1, 57	6.005	0.017	0.098	Moderate	No
Right Anterior Cingulate Gyrus	Node Degree	Musical Training	1, 57	4.122	0.047	0.070	Moderate	No
Right Mid Cingulate Gyrus	Node Degree	Musical Training	1, 57	10.751	0.002	0.164	Large	Yes
Right Mid Cingulate	Node Degree	Emotions	1, 57	4.155	0.046	0.070	Moderate	No

Brain Region	Graph Metric	Measure	*df*	*F* Value	*p* Value	Partial η^2	Effect Size	Survives Bonferroni Correction
Gyrus								
Left Thalamus	Node Degree	Emotions	1, 57	4.627	0.036	0.078	Moderate	No

Table 6: This table presents ANCOVA findings on the influence of music training, active engagement, and emotions on network metrics across selected brain regions. Data includes the targeted brain region, specific network metric, measure type, degrees of freedom (*df*), F value, p value, partial eta squared (η^2) for effect size, and the status of Bonferroni correction survivability.

Table 7 Effects of Music Perception Scores on Local Network Metrics – Controlling for Diagnosis

Brain Region	Graph Metric	Measure	*df*	*F* Value	*p* Value	Partial η^2	Effect Size	Survives Bonferroni Correction
Right Posterior Cingulate Gyrus	Clustering Coefficient	Melody	1, 67	11.803	0.001	0.154	Large	Yes
Left Hippocampus	Clustering Coefficient	Melody	1, 67	4.292	0.042	0.062	Small-Moderate	No
Right Hippocampus	Clustering Coefficient	Melody	1, 67	11.852	0.001	0.154	Large	Yes
Left Parahippocampal Gyrus	Clustering Coefficient	Melody	1, 67	5.973	0.017	0.084	Moderate	No
Left Thalamus	Clustering Coefficient	Melody	1, 67	5.736	0.020	0.081	Moderate	No
Right Thalamus	Clustering Coefficient	Melody	1, 67	4.518	0.037	0.065	Small-Moderate	No
Left Parahippocampal Gyrus	Clustering Coefficient	Melody	1, 67	4.494	0.038	0.063	Small-Moderate	No
Left Parahippocampal Gyrus	Clustering Coefficient	Beat	1, 67	4.620	0.035	0.066	Small-Moderate	No
Right Parahippocampal Gyrus	Clustering Coefficient	Beat	1, 67	5.056	0.028	0.072	Moderate	No
Left Parahippocampal Gyrus	Clustering Coefficient	Tempo	1, 67	5.624	0.021	0.080	Moderate	No
Left Thalamus	Clustering Coefficient	Tempo	1, 67	4.299	0.042	0.062	Small-Moderate	No
Right Posterior Cingulate Gyrus	Clustering Coefficient	Total mini-Proms	1, 67	7.375	0.008	0.102	Moderate	No
Left Parahippocampal Gyrus	Clustering Coefficient	Total mini-Proms	1, 67	8.497	0.005	0.116	Moderate	No
Left Thalamus	Clustering Coefficient	Total mini-Proms	1, 67	4.425	0.039	0.064	Small-Moderate	No
Right Hippocampus	Eigenvector Centrality	Melody	1, 67	6.037	0.017	0.085	Moderate	No
Left Parahippocampal Gyrus	Eigenvector Centrality	Beat	1, 67	4.362	0.041	0.063	Small-Moderate	No
Right Parahippocampal Gyrus	Betweenness Centrality	Melody	1, 67	4.683	0.034	0.067	Small-Moderate	No
Right Hippocampus	Betweenness Centrality	Tuning	1, 67	6.378	0.014	0.089	Moderate	No
Right Hippocampus	Betweenness	Beat	1, 67	8.339	0.005	0.114	Moderate	No

Brain Region	Graph Metric	Measure	*df*	*F* Value	*p* Value	Partial η^2	Effect Size	Survives Bonferroni Correction
	Centrality							
Right Hippocampus	Betweenness Centrality	Total mini-Proms	1, 67	5.905	0.018	0.083	Moderate	No
Right Mid Cingulate Gyrus	Node Degree	Melody	1, 67	6.027	0.017	0.085	Moderate	No
Left Posterior Cingulate Gyrus	Node Degree	Beat	1, 67	4.785	0.032	0.069	Small-Moderate	No
Right Hippocampus	Node Degree	Beat	1, 67	5.233	0.025	0.075	Moderate	No
Right Mid Cingulate Gyrus	Node Degree	Total mini-Proms		4.965	0.029	0.071	Moderate	No

Table 7: This table presents ANCOVA findings on the influence of music perception skills on network metrics across selected brain regions. Data includes the targeted brain region, specific network metric, measure type, degrees of freedom (*df*), F value, p value, partial eta squared (η^2) for effect size, and the status of Bonferroni correction survivability.
